# Telomere Length Dynamics and Chromosomal Instability for Predicting Individual Radiosensitivity and Risk via Machine Learning

**DOI:** 10.3390/jpm11030188

**Published:** 2021-03-08

**Authors:** Jared J. Luxton, Miles J. McKenna, Aidan M. Lewis, Lynn E. Taylor, Sameer G. Jhavar, Gregory P. Swanson, Susan M. Bailey

**Affiliations:** 1 Department of Environmental and Radiological Health Sciences, Colorado State University, Fort Collins, CO 80523, USA; Jared.Luxton@colostate.edu (J.J.L.); miles.mckenna@gmail.com (M.J.M.); aidanlew@rams.colostate.edu (A.M.L.); Lynn.Taylor@colostate.edu (L.E.T.); 2 Cell and Molecular Biology Program, Colorado State University, Fort Collins, CO 80523, USA; 3 Baylor Scott & White Medical Center, Temple, TX 76508, USA; sameer.jhavar@bswhealth.org (S.G.J.); Gregory.Swanson@bswhealth.org (G.P.S.)

**Keywords:** telomeres, chromosomal instability, inversions, prostate cancer, IMRT, machine learning, individual radiosensitivity, late effects, personalized medicine

## Abstract

The ability to predict a cancer patient’s response to radiotherapy and risk of developing adverse late health effects would greatly improve personalized treatment regimens and individual outcomes. Telomeres represent a compelling biomarker of individual radiosensitivity and risk, as exposure can result in dysfunctional telomere pathologies that coincidentally overlap with many radiation-induced late effects, ranging from degenerative conditions like fibrosis and cardiovascular disease to proliferative pathologies like cancer. Here, telomere length was longitudinally assessed in a cohort of fifteen prostate cancer patients undergoing Intensity Modulated Radiation Therapy (IMRT) utilizing Telomere Fluorescence in situ Hybridization (Telo-FISH). To evaluate genome instability and enhance predictions for individual patient risk of secondary malignancy, chromosome aberrations were assessed utilizing directional Genomic Hybridization (dGH) for high-resolution inversion detection. We present the first implementation of individual telomere length data in a machine learning model, XGBoost, trained on pre-radiotherapy (baseline) and in vitro exposed (4 Gy γ-rays) telomere length measurements, to predict post radiotherapy telomeric outcomes, which together with chromosomal instability provide insight into individual radiosensitivity and risk for radiation-induced late effects.

## 1. Introduction

Radiation late effects are a broad class of negative and often permanent health effects experienced by cancer patients long after radiation therapy [1,2], which can include cardiovascular disease (CVD) [3], pulmonary and arterial fibrosis [4], cognitive deficits [5], bone fractures [6], and secondary cancers [7]. Such late effects are of particular concern for pediatric patients [8], and risks for radiation late effects are highly dependent on patient-intrinsic factors as well, including genetics, age, sex, and lifestyle [1,2,9]. Therefore, identifying a patient’s specific risks for radiation late effects prior to radiotherapy is important for improving individual treatment planning and overall patient outcome. A number of strategies for predicting risk of developing radiation late effects have been employed, which tend to involve irradiating patient-derived samples in vitro and monitoring of biomarker(s) to infer in vivo cellular and normal tissue responses to exposure [10]; e.g., evaluation of γ-H2AX foci kinetics [11,12], apoptosis in normal blood lymphocytes [13], and chromosome aberration frequencies [14,15,16]. Additionally, Genome Wide Association Studies (GWAS) [17,18], sequencing [19], and imaging studies (i.e., radiogenomics [20]) have revealed promising putative indicators for predicting risks of late effects. However, accurately predicting an individual patient’s response to radiotherapy and associated risk of developing adverse late health effects remains challenging in terms of cost-effectiveness, throughput, and predictive power, therefore new approaches are needed.

Telomeres have been proposed as sensitive biomarkers of radiation exposure and a valuable parameter for predicting individual radiosensitivity of patients [21]. Telomeres are protective features of chromosomal termini that guard genome integrity and prevent inappropriate activation of DNA damage responses (DDRs) [22,23]. It is well established that telomeres shorten with cell division, oxidative stress [24], and aging [25]. Telomeres also shorten with a host of lifestyle factors (e.g., nutrition [26], exercise [27], stress [28]) and environmental exposures (e.g., air pollution [29], UV [30]). Telomere length is a highly heritable trait, as is telomere length regulation [31,32,33,34], supportive of individual variation in telomeric response to specific stressors. Interestingly, telomeres are regarded as hallmarks of radiosensitivity [35], and ionizing radiation (IR) exposure has been shown to evoke both shortening and lengthening of telomeres [36,37,38]. A large Mendelian randomization study [39] and recent quantitative estimates have shown that both short and long telomeres are associated with increased disease risk—of approximately equal degree [40,41]. Short telomeres are robust biomarkers and even determinants for a range of aging-related pathologies [42], including dementias, CVD and pulmonary fibrosis [43], and aplastic anemia [44], degenerative conditions also regarded as radiation late effects [45,46,47]. On the other hand, longer telomeres are associated with increased cancer risk, particularly for leukemias [48], a common cancer following IR-exposure [49]. Thus, telomere length could be used to identify radiosensitive individuals (i.e., those with shorter telomeres before radiotherapy) to better inform personalized treatment regimens. Furthermore, evaluating telomere length dynamics associated with radiotherapy could serve to identify individuals at risk for developing radiation-induced late effects; i.e., patients with shorter telomeres would be at higher risk of degenerative pathologies (fibrosis, CVD), while those with longer telomeres following radiotherapy would be at higher risk for developing proliferative pathologies, namely secondary malignancy.

Given that telomere length is influenced by a variety of genetic factors [31,32,33,34], and exposures including IR [36,37,38,50,51,52], we reasoned that a patient’s telomeric outcome post-radiation therapy, rather than their pre-treatment (baseline) measures, would be most informative for assessing individual risks for radiation late effects and long-term health consequences. Furthermore, since patient-derived pre-radiation therapy samples irradiated in vitro provide an informative proxy for individual patient radiosensitivity and response in vivo [53,54,55,56], developing an effective means to accurately predict an individual patient’s telomeric outcome post-radiation therapy would serve to improve personalized treatment strategies and individual outcomes.

Chromosome aberrations (CAs) are well-established biomarkers of IR-exposure [57] associated with virtually all cancers [58], and highly informative indicators of risk for radiation late effects, in particular, secondary cancers [14,15,16]. Ionizing radiation is exceptional in its ability to induce prompt double-strand breaks (DSBs) [59], DNA damage that necessitates a cellular response. Chromosome rearrangements result from the misrepair of such damage, and so provide a quantitative measure of cellular capacity for DNA repair [57]. In general, IR-induced CAs negatively impact cell survival and genome stability, resulting in senescence, apoptosis, and cancer [57]. Notably, chromosomal inversions and deletions have previously been proposed as signatures of radiation-induced secondary cancers [60]. Cytogenetic analysis however, is both time and labor intensive, often requiring that hundreds or even thousands of cells be scored, limiting its clinical utility [61]. We speculated that for this pilot study, inclusion of an additional type of CA, specifically inversions detected by the strand-specific methodology of directional Genome Hybridization (dGH) [62], might serve to reduce the number of cells required, while also better informing individual risk for secondary malignancy.

Significant advancements have also been made in the application of machine learning (ML) to a variety of scenarios, including predictions related to acute radiation toxicity [63], treatment planning [64], and secondary cancer risk post radiation therapy [65]. Extreme Gradient Boosting (XGBoost) is a powerful ML model that uses a gradient boosted ensemble of decision trees to learn complex relationships (linear and nonlinear) within datasets [66]. XGBoost has many translational applications, such as predicting future gastric cancer risk [67], lung cancer detection [68], and radiation-related fibrosis [69]. One potentially limiting caveat to ML is the requirement for extraordinarily large amounts of data to create robust, generalizable models. Telomere Fluorescence in situ Hybridization (Telo-FISH) is a cell-by-cell imaging-based approach for measuring telomere length capable of generating sufficient volumes of data for developing ML models; average experiments generate 200,000–1,000,000 individual telomere length measurements [70]. To date, individual telomere length measurements (Telo-FISH, Q-FISH, flow-FISH, etc.) have not been utilized in ML models for risk predictions, despite the informative nature of such an approach.

Here we provide a proof-of-principle demonstration utilizing longitudinal analysis of telomere length and chromosomal instability in fifteen (15) prostate cancer patients undergoing Intensity Modulated Radiation Therapy (IMRT). We present the first implementation of individual telomere length (Telo-FISH) data in a ML model—XGBoost—and evaluate its ability to predict post-IMRT telomeric outcomes using individual patient’s pre-IMRT (baseline) and in vitro irradiated telomere lengths. Overall, our results support use of telomere length dynamics and chromosomal instability for improved prediction of individual radiosensitivity and risk for developing radiation-induced late effects post RT.

## 2. Materials and Methods

### 2.1. Patient Consent, IMRT Therapy Information

With informed consent as per the institutional review board, 16 consecutive patients receiving pelvic and prostate or prostate fossa radiation therapy were asked to participate in the study. No patient had received androgen ablation or chemotherapy to avoid confounding factors. One patient was found to have metastatic disease after consent and was removed from further study. A total of 15 patients provided consent and blood was collected pre-IMRT (baseline), immediately post-IMRT (the last week), and 3-months post-IMRT (prior to returning to personal medical oncologist). Blood was subject to complete blood counts, and telomere length and chromosome aberration analyses. Each patient received a radiation regime consisting of 54 Gy to the pelvic lymphatics, with a total of 70 Gy (*n* = 11) or 78 Gy (*n* = 3) to the prostate fossa. One patient underwent brachytherapy boost.

### 2.2. Sample Collection and Processing for Telo-FISH and dGH

Peripheral blood was drawn and shipped in 10 mL sodium heparin tubes (Becton, Dickinson and Co, Franklin Lakes, NJ, USA; #367874) under ambient conditions to Colorado State University and received within 24 h of blood draw. All heparinized blood samples were cultured in T-25 tissue culture flasks, at 1 parts blood per 9 parts Gibco PB-Max Karyotyping Medium (Thermo Fisher, Waltham, MA, USA; #12557013), with 5.0 mM 5-bromo-deoxyuridine (BrdU) and 1.0 mM 5-bromo-deoxycytidine (BrdC) added to the medium as previously described [62]. Pre-IMRT blood samples were split into two fractions (non-irradiated and in vitro irradiated) with identical culturing conditions as other time point samples; irradiated fraction was exposed in vitro at a dose rate of 2.5 Gy/min for a total dose of 4 Gy (^137^Cs gamma-ray Mark I irradiator; J.L. Shepherd & Associates, San Fernando, CA USA). Forty-eight hours after stimulation, KaryoMax Colcemid (Thermo Fisher, Waltham, MA, USA; #15210040) was added (0.1 μg per mL of medium) for four hours of incubation, then metaphase chromosome spreads were harvested with standard cytogenetic protocols [71]. Prior to Telo-FISH and dGH, slides with metaphase chromosome spreads were subject to CO-FISH protocol for removal of BrdU/BrdC incorporated DNA as previously described [72].

### 2.3. Telomere Fluorescence In Situ Hybridization (Telo-FISH), Imaging, Quantifications

Protocol: Slides with metaphase chromosome spreads were prepared and hybridized with a fluorescently labeled telomere probe as previously described [70]. Briefly, slides were washed in 1× PBS for 5 min, dehydrated through an ice-cold ethanol series (75%, 85% and 100%) for 2 min each, air dried, and denatured in 70% formamide in 2× saline sodium citrate (SSC) at 75 °C for 2 min, followed by a second ice-cold ethanol series, and air dried again. Probe hybridization mixture consisted of G-rich (TTAGGG-′3) peptide nucleic acid (PNA) telomere probe labeled with Cyanine-3 (Cy3; Bio-synthesis, Inc., Lewisville, TX USA) at 5 nM concentration in 36 µL of formamide, 12 µL of 0.5 M Tris-HCl, 2.5 µL of 0.1 M KCl, and 0.6 µL of 0.1 M MgCl_2_. Hybridization mixture was incubated at 75 °C for 5 min and cooled on ice for 10 min, then 50 µL of mix was applied to each slide. Slides were coverslipped and hybridized at 37 °C for 4 h. After hybridization, slides were washed five times at 43.5 °C for three min each: washes one and two: 50% formamide in 2× SSC; washes three and four: 100% 2× SSC; and washes five and six: 2× SSC plus 0.1% Nonidet P-40. After washing, slides were counterstained with DAPI in Prolong Gold Antifade (Thermo Fisher, Waltham, MA, USA; #P36931), coverslipped, and stored at 4 °C for 24 h prior to imaging.

Image acquisition: Metaphase spreads (50 per patient/time point) were imaged at 100× mag on a Zeiss Axio Imager.Z2, Cool SNAP ES2 camera, and X-cite 120 LED lamp light source.

Individual telomere quantifications: Relative fluorescence intensity of individual telomeres was quantified using the ImageJ plugin Telometer [73]. Variation in Telo-FISH was controlled by assigning each patient a pair of slides made from BJ1 and BJ-hTERT fibroblast cell lines (ATCC, Manassas, VA USA). For each patient, slide preparation, Telo-FISH protocol, image acquisition and telomere quantifications were performed on the full time-course of samples and pair of BJ1/BJ-hTERT controls (50 metaphases per control) at the same time and on the same respective days. Mean telomere length was quantified for each pair of control samples yielding a ratio for standardizing patients’ telomere values as previously described [74].

### 2.4. Telo-FISH Data Processing, Feature Engineering of Short and Long Telomeres

Processing individual telomere length data: For each patient, outliers were removed from individual telomere length data per sample by omitting measurements three standard deviations from the mean; between all samples and patients, less than 1% of individual telomere measurements were considered outliers per this approach. For samples with fewer individual telomere length measurements than the theoretical number (human cells, 50 metaphase spreads), missing telomere values were imputed by randomly sampling measurements from the observed distribution of individual telomeres; randomly sampled telomeres were added up to the theoretical number of telomeres per sample.

Feature engineering short and long telomeres: Individual telomeres from the pre-IMRT non-irradiated time point were split into quartiles, designating telomeres in the bottom 25% in yellow, the middle 50% in blue, and top 25% in red. Quartile cut-off values, established by the pre-IMRT non-irradiated sample’s distribution (values that separate quartiles), were applied to subsequent time points to feature engineer the relative shortest (yellow), mid-length (blue), and longest (red) individual telomeres per time point.

### 2.5. Statistical and Clustering Analyses of Telo-FISH Data

Statistical and clustering analyses were conducted with Python in Jupyter notebooks (see Code availability). With the statsmodels library [75], mean telomere length and numbers of short and long telomeres were analyzed with a repeated measures ANOVA and post hoc Tukey’s HSD test (two-tailed *p* values for both tests). Analyses were performed on all patients (*n* = 14, less patient ID 13; 3-month post-IMRT sample failed to culture) and all four time points. A square root transformation was performed on numbers of short and long telomeres prior to statistical analysis. Ordinary least squares linear regression was performed with the scikit-learn LinearRegression tool. Hierarchical clustering analyses were performed on z-score normalized data using the scipy library [76] with a single linkage method and Pearson correlation metric. Pearson correlations between patients’ longitudinal measurements of telomere length and complete blood count data was done with Python.

### 2.6. XGBoost Models with Individual Telomere Length Data, Randomized Hyperparameter Search, Cross Validation

XGBoost models, model hyperparameter tuning, and cross validation tools were performed in Python through the scikit-learn API [77]. XGboost model features were individual telomere length values and sample labels denoted pre-IMRT sample origin (non-irradiated, in vitro irradiated), which were encoded as 0/1. Model hyperparameters were tuned using a randomized search with RandomizedSearchCV. For models predicting mean telomere length at late post-IMRT, final model hyperparameters were modified as follows: n_estimators = 200, max_depth = 7, learning_rate = 0.2, objective = ‘reg:squarederror’, random_state = 1. For models predicting short and long telomeres at late post-IMRT, final model hyperparameters were similar as for mean telomere length, with max depth = 6. Five-fold cross validation was performed with cross_val_score and a negative mean absolute error metric.

### 2.7. Directional Genomic Hybridization (dGH), Image Acquisition, Data Processing

High-resolution detection of chromosome aberrations (inversions, translocations) was performed utilizing dGH whole chromosome (Cy3) and sub-telomere (Cy5) paints specific to chromosomes 1, 2, and 3 (KromaTiD Inc., Longmont, CO USA) as previously described [62]. Briefly, slides were stained with Hoechst 33,258 (MilliporeSigma, Burlington, MA USA; #B1155) for 15 min, photolyzed for 35 min using a SpectroLinker UV Crosslinker (365 nm UV), and digested with exonuclease III (New England Biolabs, Ipswich, NA, USA; #M0206L) for 30 min. Paint hybridization mixture was applied to slides, which were then coverslipped, sealed with rubber cement, and denatured at 70 °C for three min. Slides were hybridized for 24 h at 37 °C, followed by five washes in 2× SSC at 43.5 °C. After washing, slides were counterstained with one drop of DAPI in Prolong Gold Antifade (Thermo Fisher, Waltham, MA, USA; #P36931), coverslipped, and stored at 4 °C for 24 h prior to imaging. Metaphase spreads (30 per patient/time point) were imaged/scored at 63× mag on a Zeiss Axio Imager.Z2, Cool SNAP ES2 camera, and X-cite 120 LED lamp light source. Counts of chromosome aberrations were corrected for clonality, where identical aberrations between cells for a patient’s given time point were noted but scored only once.

### 2.8. Statistical and Clustering Analyses of Chromosome Aberrations (dGH)

Statistical and clustering analyses were conducted with Python in Jupyter notebooks (see Code availability). With the statsmodels library, average chromosome aberration frequencies were analyzed with a repeated measures ANOVA and post hoc Tukey’s HSD test (two-tailed *p* values for both tests). Analyses were performed on all patients (*n* = 14, less patient ID 13; 3-month post-IMRT sample failed to culture) and all-time course samples (4). Ordinary least squares linear regression was performed with the scikit-learn LinearRegression tool. Hierarchical clustering analyses were performed on z-score normalized data using the scipy library with a single linkage method and Pearson correlation metric. Pearson correlations between patients’ longitudinal measurements of average chromosome aberration frequencies and complete blood count data was done with Python.

### 2.9. XGBoost Model Design with Chromosome Aberrations

XGBoost models, model hyperparameter tuning, and cross validation were accessed in Python via the same manner as described for Telo-FISH data above. XGboost model features were counts of scored chromosome aberrations per cell, with sample labels denoting pre-IMRT sample origin (non-irradiated, in vitro irradiated; encoded as 0/1). Model hyperparameters were tuned using a randomized search with RandomizedSearchCV; models were ultimately non-performant. Final model hyperparameters (used with all chromosome aberrations) were: n_estimators = 200, max_depth = 15, learning_rate = 0.1, objective = ‘reg:squarederror’, random_state = 0. Five-fold cross validation was performed with a negative mean absolute error metric. 

## 3. Results

### 3.1. Longitudinal Analyses of Telomere Length Associated with Radiation Therapy

**Figure 1:**
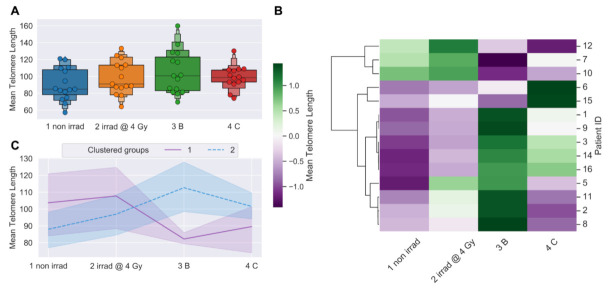
Telomere length dynamics (Telo-FISH). Mean telomere length expressed as relative fluorescence intensity. (**A**) Time-course of blood sample collection for all prostate cancer patients (*n* = 15; 50 cells/patient/time point scored): 1 non irrad = pre-IMRT non-irradiated (0 Gy); 2 irrad @ 4 Gy: pre-IMRT in vitro irradiated; 3B: immediate post-IMRT; and 4C: 3-months post-IMRT. Boxes denote quantiles, horizontal grey lines denote medians. Telomere length values were standardized using BJ1/BJ-hTERT controls. (**B)** Hierarchical clustering of patients by longitudinal changes in mean telomere length (z-score normalized). (**C**) Time-course for clustered groups of patients (*n* = 3, purple; *n* = 11, blue); center lines denote medians, lighter bands denote confidence intervals. Patient ID 13 not clustered (sample failed to culture). Significance was assessed using a repeated measures ANOVA and post hoc Tukey’s HSD test.

Blood was collected from 15 prostate cancer patients undergoing IMRT at baseline (pre-IMRT), immediately post-IMRT (conclusion of treatment regimen), and 3-months post-IMRT. Baseline blood samples were split, half serving as the non-irradiated control (0 Gy), and the other half irradiated in vitro (4 Gy, Cs^137^ γ-rays) as a proxy for individual radiation response [55].The lengths of thousands of individual telomeres (*n* = 50 cells/patient/time point) were measured on metaphase chromosomes (lymphocytes stimulated from whole blood) by Telo-FISH at all time points [pre-therapy non-irradiated (0 Gy); pre-therapy in vitro irradiated (4 Gy); immediately post-IMRT; and 3-months post-IMRT] (Figure 1A). For the overall cohort, differences in mean telomere length (MTL) between samples approached but did not reach statistical significance (*p* = 0.059, repeated measures ANOVA). Relative to the pre-IMRT non-irradiated samples (0 Gy), overall MTL modestly increased after 4 Gy in vitro irradiation, and showed an even greater increase immediately after completion of the IMRT regimen, suggesting that increased MTL is an overall response to radiation exposure in this cohort. At 3-months post-IMRT, MTL for the cohort approached pre-IMRT levels.

Complete blood counts (CBC) were evaluated in the same samples, and longitudinal changes in patients’ MTL negatively correlated (R^2^ = −0.126) with total peripheral white blood cell (WBC) counts (Figure S1A). Longitudinal correlations between numbers of WBC types and MTL (all time points, for each patient) revealed a positive relationship with basophils (R^2^ = 0.278) and a negative relationship with lymphocytes (R^2^ = −0.294) (Figure S1B). Furthermore, longitudinal correlations between MTL and the proportions of lymphocyte sub-groups (all time points, for each patient) revealed positive relationships with natural killer (NK) and CD4 cells (R^2^ = 0.408, 0.282), and negative relationships with CD8 and CD19 cells (R^2^ = −0.251, −0.288) (Figure S1C). These results support the notion that the overall changes in MTL associated with radiation exposure, specifically apparent telomere elongation, could be at least partially due to cell killing and shifts in lymphocyte populations, as previously proposed [78,79].

### 3.2. Telomere Length Dynamics Revealed Individual Differences in Radiation Response

We hypothesized that groups of patients would cluster based on differential telomeric responses to radiation therapy, with sub-groups displaying either shorter or longer MTL post-IMRT. Clustering patients by longitudinal changes in MTL revealed two broad trends over time (Figure 1B). Patients that clustered in group 1 (*n* = 3) had relatively longer MTL at baseline (pre-IMRT), and showed a dramatic, persistent decrease in MTL post-IMRT (Figure 1C). Those patients that clustered in group 2 (*n* = 11) had relatively shorter MTL at baseline, and showed a dramatic, sustained increase in MTL post-IMRT (Figure 1C). Reduced MTL 3-months post-IMRT suggests increased risks for degenerative radiation late effects [43], while increased MTL suggests increased risks for proliferative secondary cancers [48].

**Figure 2:**
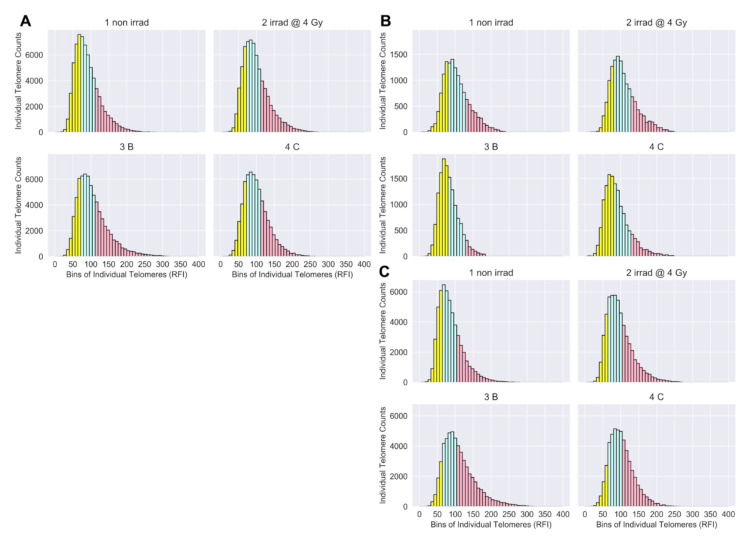
Telomere length distributions (Telo-FISH). Individual telomere length distributions of prostate cancer patients (*n* = 15): 1 non irrad = pre-IMRT non-irradiated (0 Gy); 2 irrad @ 4 Gy = pre-IMRT in vitro irradiated; 3B = immediate post-IMRT; and 4C = 3-months post-IMRT. RFI: Relative Fluorescence Intensity. Individual telomeres from the pre-therapy non-irradiated time point were split into quartiles, designating telomeres in the bottom 25% (yellow), middle 50% (blue), and top 25% (red). Quartile cut-off values, established by the distribution of the pre-therapy non-irradiated time point, were applied to subsequent time points to feature engineer the relative shortest, mid-length, and longest individual telomeres per time point. (**A**) Individual telomere length distributions for all patients (averaged) per time point. (**B**) Individual telomere length distributions for patients in mean telomere length clustered group 1 (*n* = 3) and (**C**) group 2 (*n* = 11).

In addition to MTL, Telo-FISH provides measures for many hundreds of individual telomeres, enabling generation of telomere length distributions and longitudinal analysis of shifts in populations of short and long telomeres [70]. For the overall cohort, numbers of short telomeres decreased and numbers of long telomeres dramatically increased 3-months post-IMRT (Figure 2A). When individual telomeres from patients in the MTL clustered group 1 (*n* = 3) were combined, dramatic and persistent increases in the numbers of short telomeres post-IMRT were observed (Figure 2B), while MTL clustered group 2 patients (*n* = 11) showed dramatic and persistent increases in numbers of long telomeres post-IMRT (Figure 2C). Again, patients with increased numbers of short telomeres are presumed to have increased risks for degenerative radiation late effects [43], while those with increased numbers of long telomeres are at increased risk of secondary cancers [48]. Numbers of short and long telomeres were feature engineered (see Materials and Methods) from each patient’s individual telomere length data for further analysis.

**Figure 3:**
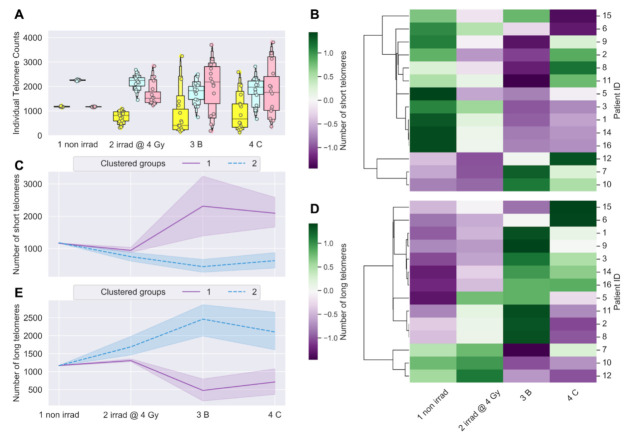
Longitudinal shifts in numbers of short and long telomeres (Telo-FISH). Numbers of short and long telomeres from individual telomere length distributions: 1 non irrad = pre-IMRT non-irradiated (0 Gy); 2 irrad @ 4 Gy = pre-IMRT in vitro irradiated; 3B = immediate post-IMRT; and 4C = 3-months post-IMRT. Shortest (yellow), mid-length (blue), and longest (red) telomeres were feature engineered per patient (*n* = 15). (**A**) Counts of short, medium, and long telomeres; 4600 individual telomeres per patient per time point. Significance was assessed using a square-root transformation and a repeated measures ANOVA with post hoc Tukey’s HSD test. Hierarchical clustering of patients by longitudinal changes in numbers of short (**B**) and long telomeres (**D**) (z-score normalized). Time-courses of patient groups (*n* = 3, purple; *n* = 11, blue) clustered by numbers of short (**C**) and long (**E**) telomeres; center lines denote medians and lighter bands denote confidence intervals. Patient ID 13 not clustered (sample failed to culture).

Differences in the average number of short and long telomeres between samples approached but did not reach statistical significance for the overall cohort (*p* < 0.1; repeated measures ANOVA) (Figure 3A). We speculated that clustering patients by numbers of short or long telomeres would reveal longitudinal trends similar to those observed when clustering patients by MTL (Figure 1B,C). Clustering patients by longitudinal changes in numbers of short or long telomeres (Figure 3B,D) revealed two broad trends over time (Figure 3C,E). Clustered group 1 (*n* = 3) showed a dramatic, sustained increase in numbers of short telomeres post-IMRT, with a corresponding decrease in numbers of long telomeres (Figure 3C,E). Clustered group 2 (*n* = 11) showed a dramatic, nearly uniform decrease in numbers of short telomeres post-IMRT, with a corresponding increase in long telomeres (Figure 3C,E). Importantly, clustering patients either by MTL or by numbers of short or long telomeres post-IMRT identified the same three patients with shorter telomeres, and eleven with longer telomeres (Figure 1B and Figure 3B,D).

### 3.3. Linear Regression Failed to Predict Post-IMRT Telomeric Outcomes

**Figure 4:**
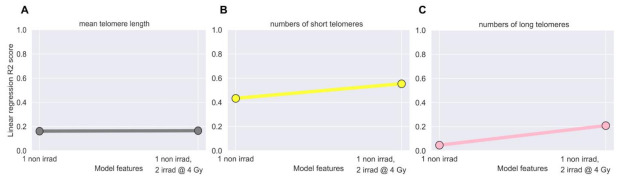
Linear regression models failed to predict post-IMRT telomeric outcomes. Ordinary least squares linear regression models were employed using pre-IMRT telomeric data (Telo-FISH) from the pre-IMRT non-irradiated (0 Gy) or the pre-IMRT in vitro irradiated (4 Gy) samples to predict 3-month post-IMRT telomeric outcomes. Models were made using (**A**) mean telomere length (R^2^ = 0.161, 0.165), (**B**) numbers of short (R^2^ = 0.433, 0.554), and (**C**) numbers of long (R^2^ = 0.046, 0.208) telomeres.

Based on the two distinct groups identified by MTL and numbers of short and long telomeres 3-months post-IMRT (Figure 1 and Figure 3), we hypothesized that pre-IMRT measurements of MTL and numbers of short and long telomeres could predict their respective post-IMRT outcomes using linear regression. For MTL, two linear regression models were created. The first used only MTL from pre-IMRT (baseline) non-irradiated samples as the independent variable, and the second used MTL from both the non-irradiated and in vitro irradiated pre-IMRT samples as independent variables for predicting post-IMRT MTL (Figure 4A). The R^2^ values for the two models were 0.161 and 0.165, respectively (Figure 4A), evidence that linear regression poorly captured the relationship between pre- and post-IMRT MTL. For numbers of short and long telomeres, two linear regression models were similarly created. The models for short telomeres yielded R^2^ values of 0.433 and 0.554, and the models for long telomeres yielded R^2^ values of 0.046 and 0.208 (Figure 4B,C). While the models for numbers of short telomeres had modestly higher R^2^ values than those for MTL or long telomeres, all linear regression models performed too poorly to confidently predict telomeric outcomes.

### 3.4. Development of XGBoost Machine Learning Models for Accurate Prediction of Post-IMRT Telomeric Outcomes

The fact that linear regression poorly predicted post-IMRT telomeric outcomes could be due to the low number of observations (*n* = 14), and/or the nonlinearity of telomere length dynamics (changes over time) in response to radiation exposure (Figure 1, Figure 2, Figure 3 and Figure 4). We sought an alternative approach that could effectively utilize our vast dataset of pre-IMRT individual telomere length measurements (*n* = 128,800), and also capture the nonlinearity of telomeric responses. Considering that XGBoost had recently been used to predict cancer risk and radiation-induced fibrosis using patient data [66,67,68,69], we hypothesized that XGBoost models could be trained with pre-IMRT individual telomere length measurements to accurately predict post-IMRT telomeric outcomes.

**Figure 5:**
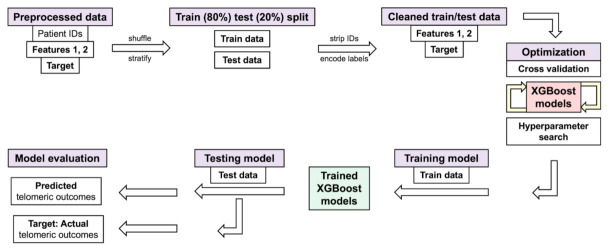
Processing of Telo-FISH data for training and testing XGBoost models. Schematic for machine learning pipeline used for individual telomere length data (Telo-FISH). Preprocessed data: Feature 1: pre-IMRT individual telomere length measurements (*n* = 128,800); Feature 2: pre-IMRT sample labels (non-irradiated, in vitro irradiated, encoded as 0/1); Target: 3 months post-IMRT telomeric outcomes (mean telomere length or numbers of short and long telomeres). Data is randomly shuffled and stratified (by patient ID and pre-therapy sample origin) and split into training (80%) and test (20%) datasets; patient IDs are stripped after splitting. Five-fold cross validation was used, and models were evaluated with Mean Absolute Error (MAE) and R^2^ scores between predicted and true values in the test set.

Pre-IMRT (baseline) telomere length data required extensive processing prior to training the XGBoost model for predicting three-month post-IMRT MTL (Figure 5). Data was reshaped into a matrix consisting of 128,800 rows (one for each individual telomere measurement) and four columns: patient ID, individual telomere length value, label denoting pre-IMRT sample of origin (non-irradiated or in vitro irradiated), and three-month post-IMRT MTL (Table S1A). Reshaped data was randomly shuffled and stratified by patient ID and sample of origin, then split into training (80% of total) and test (20% of total) datasets. Shuffling guarded against order of measurement bias (Telo-FISH image acquisition), while stratifying ensured equivalent numbers of individual telomeres from each patients’ pre-IMRT samples (non-irradiated vs. in vitro irradiated) in the training and test datasets. Patient IDs were stripped from the training and test datasets, and individual telomeres from the non-irradiated and in vitro irradiated samples were encoded as 0 and 1 to denote sample origin (Table S1B). XGBoost model hyperparameters were optimized using a randomized hyperparameter search [80].

**Figure 6:**
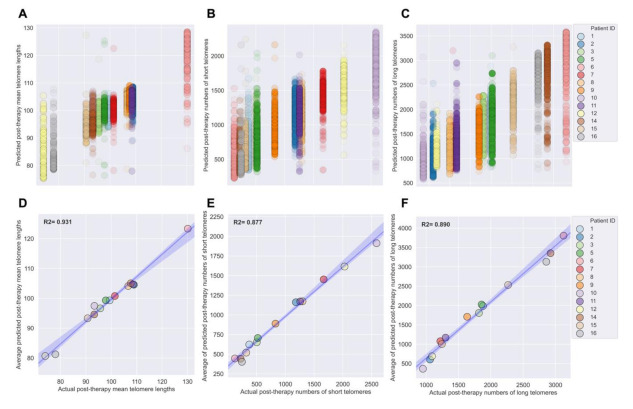
High performance of XGBoost models for predicting post-IMRT telomeric outcomes. Three separate XGBoost models were trained on pre-IMRT individual telomere length measurements (*n* = 103,040, Telo-FISH) to predict 3-month post-IMRT telomeric outcomes. Trained XGBoost models were challenged with the test set (new data, *n* = 25,760 individual telomeres) to predict 3-month post-IMRT telomeric outcomes for (**A**) mean telomere length, (**B**) numbers of short, and (**C**) numbers of long telomeres. XGBoost predictions were averaged on a per patient basis for (**D**) mean telomere length, (**E**) numbers of short, and (**F**) numbers of long telomeres; blue line represents a simple regression line (X/Y), lighter bands the 95% confidence interval, R^2^ values (coefficient of determination) are noted in bold.

XGBoost model performance was evaluated across the training dataset using five-fold cross validation [81]. Mean absolute error (MAE), the mean of all differences between predicted and actual values of mean telomere length, was used to assess the model’s performance and ability to generalize to new data (Table S2A). Five-fold cross validation on the full training dataset yielded an average MAE of 3.233 with a standard deviation of 0.052 (Table S2A), suggesting that the model was not overfitting to portions (folds) of the training data and that it could generalize to new data. Model performance was also evaluated when training across variable numbers of individual telomere measurements (*n* = 100 to 103,040) (Table S2A). After training the XGBoost model on the full training dataset, the model was challenged to predict three-month post-IMRT MTL using new data—the test dataset. The XGBoost model predictions for MTLs in the test set matched the true values with an R^2^ value of 0.882 (Figure 6A; Table S2A). Averaging predictions per patient for three-month post-IMRT MTL in the test set increased the R^2^ value to 0.931 (Figure 6D).

**Figure 7:**
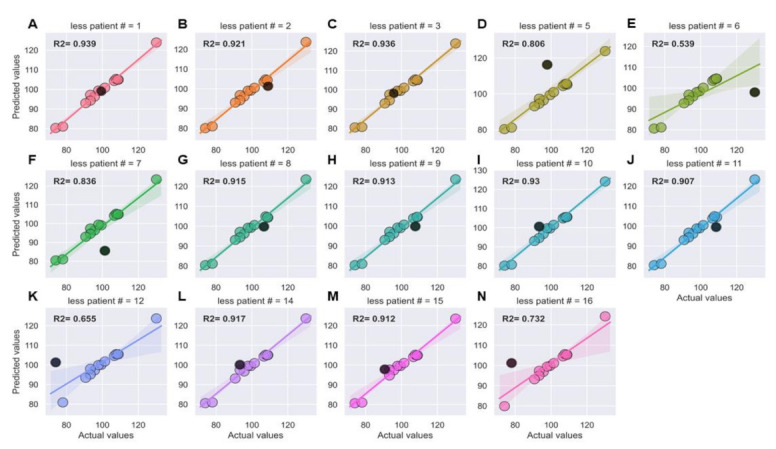
Strong generalizability of XGBoost models to new patient data (leave one out approach). (**A**–**N**) Fourteen separate XGBoost models were iteratively trained on pre-IMRT individual telomere length measurements (*n* = 93,840, Telo-FISH) excluding one patient, and tested to predict 3-month post-IMRT mean telomere length, with inclusion of the patient excluded during training. Each panel is one model; patients excluded during training for that model are noted in the panel headers and plotted in black. Lines represent a simple regression line (X/Y), lighter bands the 95% confidence interval, R^2^ values (coefficient of determination) are noted in bold.

XGBoost models were then challenged to predict post-IMRT telomere length of patients whose pre-IMRT individual telomeres they were not trained on. We iteratively trained 14 XGBoost models, where in each model one patient’s individual telomeres were “left out” of the training (i.e., round robin approach), but were included during model testing. Generally speaking, the XGboost models were extremely performant in predicting post-IMRT telomere length on entirely new patients (Figure 7A–N). Some deviations in performance were observed—we attribute these deviations in performance to low sample size. We also attempted a “leave two” and “leave three” patients out training and testing approach to understand the limits of generalizability for our XGBoost models (Figures S2A–M and S3A–L). We again found evidence of strong generalizability of the models to new patients. Together, these results demonstrate that the XGBoost model learned the nonlinear relationships between pre-IMRT individual telomere length data and three-month post-IMRT MTLs (training dataset), and also generalized to new data (test dataset) and new patients with highly accurate predictions.

Pre-IMRT individual telomere length data was also processed and reshaped for training separate XGBoost models to predict numbers of short or long telomeres 3-months post-IMRT (Figure 5, Table S1C,E). Reshaped data was split into training (80%) and test (20%) datasets and shuffled and stratified in an identical manner as described for MTL (Table S1D,F). Hyperparameters of the XGBoost models were optimized using a randomized search [80], and the models performance and generalizability were analyzed using five-fold cross validation [81] with a MAE error metric. For XGBoost models of short telomeres, five-fold cross validation on the full training dataset yielded an average MAE of 236.283 with a standard deviation of 2.059 (Table S2B), while XGBoost models of long telomeres yielded an average MAE of 330.352 and standard deviation of 2.086, suggesting that both models were reasonably good at fitting the data and likely to generalize to new data (Table S2C). Model performance was also evaluated using variable numbers of training data (*n* = 100 to 103,040). Fully trained XGBoost models were challenged with predicting three-month post-IMRT numbers of short or long telomeres in the test set, and predictions matched the true values with an R^2^ value of 0.811 and 0.819, respectively (Figure 6B,C; Table S2B,C). Averaging predictions per patient for post-IMRT numbers of short or long telomeres increased the R^2^ value to 0.877 and 0.890, respectively (Figure 6E,F). These results demonstrate that the XGBoost models learned the relationships between pre-IMRT individual telomere length data and three-month post-IMRT numbers of short or long telomeres (training dataset), and effectively generalized to new data (test dataset).

### 3.5. Longitudinal Analyses of Chromosomal Instability Associated with Radiation Therapy

**Figure 8:**
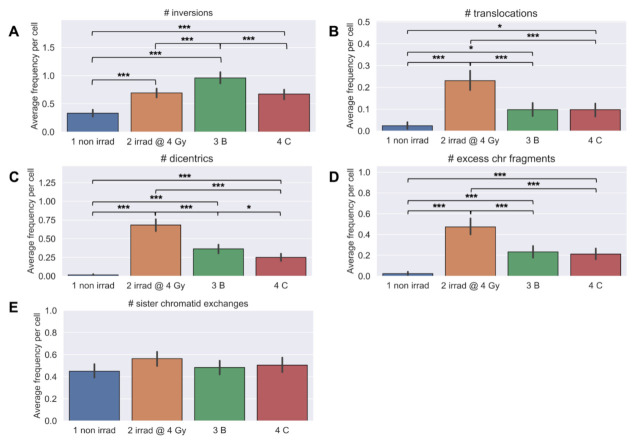
Longitudinal analyses of chromosomal instability. Whole blood was collected from prostate cancer patients undergoing IMRT (*n* = 15) and chromosome aberrations assessed using directional Genomic Hybridization (dGH) on metaphase spreads (*n* = 30/patient/timepoint scored): 1 non irrad = pre-IMRT non-irradiated (0 Gy); 2 irrad @ 4 Gy = pre-IMRT in vitro irradiated; 3B = immediate post-IMRT; and 4C = 3-month post-IMRT. Frequencies of (**A**) inversions, (**B**) translocations, (**C**) dicentrics, (**D**) excess chromosome fragments (deletions), and (**E**) sister chromatid exchanges (SCE). Significance was assessed for average aberration frequencies using a repeated measures ANOVA and post hoc Tukey’s HSD test. *p* < 0.05 *, *p* < 0.01 **, *p* < *0*.001 ***.

Directional Genomic Hybridization (dGH) is a cytogenomics-based methodology for high-resolution detection of chromosome aberrations (CAs), including structural variants missed even by sequencing [82], particularly inversions [62,78]. We hypothesized that including inversions would facilitate scoring fewer metaphase spreads (*n* = 30/time point/patient) [61], while also improving evaluation of individual chromosomal instability, and thus the ability to infer patients at higher risks for secondary cancers. Many significant differences in frequencies of IR-induced rearrangements were observed (Figure 8A–D), with inversions occurring at the highest frequencies, consistent with expectations [62,78]. Interestingly, overall average frequencies of inversions at 3-months post-IMRT were comparable to the in vitro irradiated samples (Figure 8A). Frequencies of translocations, dicentrics, and excess chromosome fragments (deletions) were highest after in vitro irradiation, and were also significantly elevated immediately post-IMRT (Figure 8B–D). Significantly elevated frequencies of translocations, dicentrics, and chromosome fragments persisted 3-months post-IMRT (Figure 8B–D). Frequencies of sister chromatid exchanges (SCE) did not significantly change over time (Figure 8E), consistent with expectation and low linear energy transfer (LET) radiation exposure [83,84]. Taken together, significantly elevated frequencies of CAs 3-months post-IMRT confirmed genomic instability in the cohort [57,58,60].

Significant changes in frequencies of IR-induced rearrangements also correlated with numbers of peripheral blood lymphocytes. Longitudinal correlations between patients’ average frequencies of CAs and numbers of peripheral blood lymphocytes (all time points) revealed strongly negative correlations (Figure S4A–D). Frequencies of inversions and dicentrics had the highest negative correlations (R^2^ = −0.752, −0.751), indicating they were highly informative—and similar—markers for cell death. These results suggest that patients demonstrating chromosomal instability (specifically, elevated frequencies of inversions and/or dicentrics), also experience higher levels of cell killing (i.e., greater radiosensitivity) consistent with previous reports [85,86].

**Figure 9:**
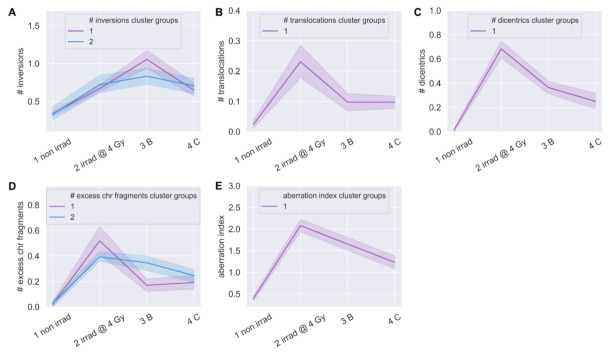
Clustering of patients by chromosome aberration frequencies. Time-courses for groups of patients hierarchically clustered into discrete groups (blue, purple) per aberration type: 1 non irrad = pre-IMRT non-irradiated (0 Gy); 2 irrad @ 4 Gy = pre-IMRT in vitro irradiated; 3B = immediate post-IMRT; and 4C = 3-month post-IMRT. Clustered groups of patients for frequencies of (**A**) inversions, (**B**) translocations, (**C**) dicentrics, (**D**) excess chromosome fragments (deletions), and (**E**) aberration index, which was created by summing all aberration types. Center lines denote medians and lighter bands denote confidence intervals.

Next, we hypothesized that clustering patients by longitudinal changes in CA frequencies (all samples) would reveal groups of patients with lower or higher frequencies of CAs, which would be indicative of individual chromosomal instability and radiosensitivity. When clustering patients by CA type, we observed groups of patients with differential responses only for inversions and excess chromosome fragments (deletions), which displayed increased frequencies immediately post-IMRT (Figure 9A,D, Figure S5A,B). We note that the two patients with the highest post-IMRT frequencies of inversions (ID #16) and chromosome fragments (ID #6), also had very high post-IMRT MTLs, supportive of correlation between these informative biomarkers, and suggestive of increased risks for secondary cancers [14,15,16,48] (Figure 1C and Figure 9A,D, Figure S5A,B).

Other CA types presented longitudinal responses that were relatively similar between patients and did not cluster patients (Figure 9B,C and Figure S6A,B). We hypothesized that while individual types of CAs failed to cluster patients into groups, individual patients may show lower or higher frequencies of CAs. To determine if some patients showed a general susceptibility to chromosomal instability, we feature engineered an ‘aberration index’ by summing all types of CAs (less sister chromatid exchanges) (Figure 9A–D) per cell for all time points. As indicated by the aberration index, groups of patients with lower or higher total CA frequencies were not observed (Figure 9E and Figure S6C). These results, together with the telomere length data, identified two patients (ID #s 6, 16) at potentially increased risks for secondary cancers [14,15,16,48], and are supportive of inversions and deletions being more informative than other CA types for predicting IR-induced secondary cancers, consistent with previous report [60]. While results indicate that the numbers of cells scored were too low (*n* = 30) to detect significant differences in individual patient susceptibility to chromosomal instability in general, including inversions improves power on an individual basis.

### 3.6. Linear Regression Poorly Predicted Radiation-Induced Chromosomal Instability

**Figure 10:**
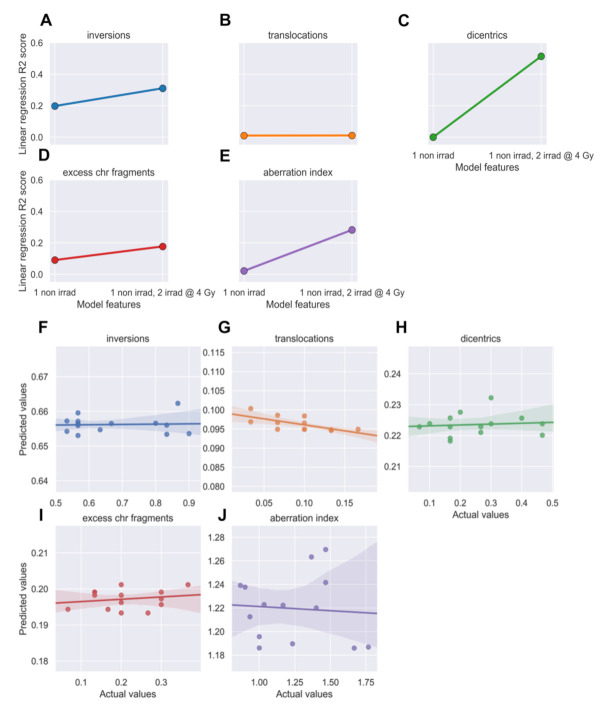
Neither linear regression nor XGBoost models successfully predicted post-IMRT chromosome aberration (CA) frequencies. Ordinary least squares linear regression models were made using pre-IMRT average CA frequencies from the non-irradiated (0 Gy) or in vitro irradiated (4 Gy) samples to predict 3-month post-IMRT average CA frequencies. Models were made for (**A**) inversions, (**B**) translocations, (**C**) dicentrics, (**D**) excess chromosome fragments (deletions), and (**E**) aberration index, which was created by summing all CA per cell. The model for dicentrics performed best, with an R^2^ = 0.514. XGBoost models were trained on pre-IMRT counts of different CA types per cell (*n* = 672) to predict 3-month post-IMRT average CA frequencies. Trained XGBoost models were challenged with the test set (new data, *n* = 168 cells) to predict 3-month post-IMRT average CA frequencies. XGBoost predictions were averaged on a per patient basis for (**F**) inversions, (**G**) translocations, (**H**) dicentrics, (**I**) excess chromosome fragments (deletions), and (**J**) aberration index. For all models, R^2^ values between averaged predictions and actual values did not exceed 0.100.

We speculated that pre-IMRT CA frequencies could be predictive of post-IMRT frequencies. Two linear regression models were made for each CA type to predict post-IMRT frequencies; the first used only the pre-IMRT (baseline) non-irradiated sample CA frequency, and the second used CA frequencies from both pre-IMRT non-irradiated and in vitro irradiated samples. The models showed poor predictive power overall, and although inclusion of the in vitro irradiated sample data improved performance overall, both models were insufficient for predicting post-IMRT CA frequencies with confidence (Figure 10A–E). The model for dicentrics performed best, with an R^2^ score of 0.514 when using data from both irradiated and non-irradiated baseline samples. These results suggest that while in vitro irradiated sample data added predictive power, the number of cells scored per time point/patient (*n* = 30) was too low to enable accurate predictions of individual patient outcomes regarding CAs frequencies post-IMRT using linear regression.

### 3.7. XGBoost Machine Learning Models Poorly Predicted Radiation-Induced Chromosomal Instability

We attempted training XGBoost models using pre-IMRT (baseline) CA data to predict post-IMRT CA frequencies. Rather than using CA data per patient, which would be insufficient for model training (*n* = 15), we used pre-IMRT CA frequencies on a per cell basis (*n* = 840) to predict three-month post-IMRT average CA frequencies. Pre-IMRT CA frequency data was extensively processed prior to XGBoost model training (Figure S7; Table S3A–D), in a nearly identical manner as described for pre-IMRT telomere length data. The key difference was that CA data was reshaped to train XGBoost models with pre-IMRT CA count data per cell (*n* = 672 cells) in order to predict three-month post-IMRT average CA frequencies. Separate datasets and XGBoost models were created for each type of CA (see Materials and Methods).

XGBoost models for each CA type were evaluated across their respective training sets using five-fold cross validation [81] with a MAE metric. The cross-validation metrics for all XGBoost models with CA data suggested a failure of the models to learn relationships between pre-IMRT CA count data per cell and three-month post-IMRT average CA frequencies (Table S4A). Furthermore, dramatic fluctuations in model performance were noted when running multiple iterations of cross-validation, again suggesting that the models failed to learn the relationships between the pre- and post-IMRT CA frequencies (Table S4A–C). We attempted to improve model performance with many types of feature engineering (e.g., Boolean features), numerical transformations, and adjustments to model hyperparameters, none of which yielded meaningful improvements in any combination (data not shown). Regardless of poor model performance in cross-validation, we challenged the XGBoost models to predict post-IMRT average CA frequencies using pre-IMRT CA count data per cell in the test set (*n* = 168 cells). In XGBoost model predictions for 3-month post-IMRT CA frequencies in the test set, none of the predictions matched the true values, with an R^2^ above 0.1 (Figure 10F–J, Table S4A–C). These results indicate that either the amount of data was insufficient for training XGBoost models (*n* = 840 cells at pre-IMRT), or the strategy of predicting post-IMRT average CA frequencies using pre-IMRT CA count data per cell was inherently faulty.

## 4. Discussion

The response of patients to radiotherapy varies considerably. Thus, it is important to identify radiosensitive individuals, as well as those most at risk of developing adverse late effects. Better prediction of a cancer patient’s individual response to radiation therapy and risk for developing adverse late health effects remains a prime objective for the treatment modality in general [1,2,3,4,5,6,7], and particularly in regard to pediatric patients [8]. Over recent years, a variety of approaches for predicting radiation late effects have been developed [10,11,12,13,14,15,16,17,18,19,20], albeit with varying degrees of compromise between cost-effectiveness, throughput, and predictive power. One particularly promising exception is the use of ML models, which can leverage extensive amounts of patient data to make accurate predictions of treatment outcomes [63,64,65,67,68,69].

Predicting a patient’s telomeric response to radiation therapy is of clinical interest for predicting risks of radiation late effects, as shorter telomeres confer radiosensitivity [35] and increase the risk of degenerative late effects (CVD and pulmonary fibrosis (Martínez and Blasco, 2018), aplastic anemia [44]), while longer telomeres increase risk for secondary cancers, particularly leukemias [48]. Given that telomeric responses to radiation exposure can be highly dynamic [36,37,38,50,51,52] and vary between individuals (Figure 1, Figure 2 and Figure 3), a framework for predicting a patient’s particular telomeric responses to radiation therapy is essential for utilizing telomere length as an informative biomarker of radiation late effects and secondary cancers. Here, we demonstrate the feasibility of using ML to accurately predict an individual patient’s telomeric response to radiation therapy. We successfully implemented individual telomere length data in a machine learning model, XGBoost, for highly accurate predictions of post-IMRT telomeric outcomes (Figure 5 and Figure 6; Table S3). The ML models and Telo-FISH methods used are fully available, providing a valuable resource for continued research into telomere length as a biomarker for radiation late effects associated with any manner of exposure.

The possibility of improving assessment of chromosomal instability and associated risk for development of secondary cancers following radiation therapy [14,15,16] was explored utilizing dGH, which facilitated inversion detection at higher resolution than traditional cytogenetic assays [62,78]. Indeed, inversions were observed at higher frequencies than other types of CAs both before and after radiation therapy (Figure 8A), consistent with prior reports [62,78]. Groups of patients with increased frequencies of chromosomal inversions and excess fragments (deletions), previously proposed signatures of radiation-induced cancers [60], were also observed 3-months post-IMRT (Figure 9A,D). Two patients from these groups had very high MTLs 3-months post-IMRT as well, also supportive of increased risks for secondary cancers [14,15,16,48]. We attempted to derive some predictive value from CA data with linear regression and XGBoost implementations, however, neither approach was successful. Even with the inclusion of inversions, the low numbers of cells scored per patient likely subverted successful predictions from the data.

Although we were unable to predict post-IMRT CA frequencies, the strong correlations between patients’ average frequencies of CAs and changes in peripheral blood lymphocyte counts associated with IMRT supported the value of inversions for evaluating chromosomal instability. Inversions and dicentrics in particular had strong, negative correlations with lymphocyte cell counts (R^2^ = −0.752, −0.751) (Figure S1A,B). Thus, patients with higher levels of radiation therapy-induced chromosomal instability also experienced increased levels of cell death, i.e., they exhibited individual radiosensitivity [85,86].

Relationships between peripheral blood cell count data and MTL were also observed. Counts of peripheral WBCs were negatively correlated with MTL associated with IMRT (R^2^ = −0.126), supportive of shorter telomeres contributing to cell killing and individual radiosensitivity (Figure S1A). When parsing WBCs by sub-type, a stronger negative relationship between MTL and lymphocyte counts was seen (R^2^ = −0.294). When parsing lymphocytes by sub-type and correlating MTL with the proportions of cell-types, positive correlations with NK and CD4 cells (R^2^ = 0.408, 0.282), and negative correlations with CD8 and CD19 cells (R^2^ = −0.251, −0.288) were observed. These results support our previously proposed supposition that the observed changes in MTL associated with radiation exposure could be at least partially due to cell killing and associated changes in peripheral blood lymphocyte cell populations [78,79].

Longitudinal assessment of individual telomere lengths by Telo-FISH in cancer patients undergoing IMRT facilitated demonstration of XGBoost as the ML model of choice for predicting telomeric outcomes post-IMRT. Given the notion that risks for radiation late effects occur on a spectrum [1,2,3,4,5,6,7,8], and the differential telomeric responses between individuals and radiation modalities, we posit that the true range of telomeric responses for radiation therapy patients in general is much broader than those observed here in this prostate cancer cohort (Figure 1, Figure 2 and Figure 3). Thus, while our XGBoost models effectively generalized to new data within our experimental design (similar patient sex, radiation modality, cancer type, etc.), it is unlikely that our trained models, in their current iteration, would generalize to data collected under different experimental or clinical parameters. Moreover, with regard to measurement of individual telomere lengths for training XGBoost models, Telo-FISH could readily be interchanged with comparable assays (Q-FISH, flow-FISH), which would provide higher throughput. Additionally, the ML approaches described here were not strictly dependent upon XGBoost, and could be conducted using other machine learning models and frameworks (e.g., random forests, kNN). Our paradigm of training ML models with individual telomere length data for prediction of post-IMRT telomeric outcomes provides improved predictive power and novel insight into individual radiosensitivity and risk of radiation-late effects, as well as a general framework that could be implemented for radiation therapy patients regardless of cancer type, radiation modality, or individual patient sex or genetic susceptibilities.

## Data Availability

Raw and processed individual telomere length (Telo-FISH) data files and chromosome aberration score sheets (dGH) are available for download at https://github.com/Jared-Luxton/. All data processing pipelines and code were written in Python and stored in Jupyter notebooks at https://github.com/Jared-Luxton/. The Jupyter notebooks can be run within a web browser and are available for download.

## Supplementary Materials

The following are available online at https://www.mdpi.com/2075-4426/11/3/188/s1, Figure S1. Correlations between telomere length, peripheral white blood cells, and lymphocytes, Figure S2. Strong generalizability of XGBoost models to new patient data (leave one out), Figure S3. Strong generalizability of XGBoost models to new patient data (leave two out), Figure S4. Correlations between chromosome aberrations and lymphocyte cell counts, Figure S5. Clustering of patients by inversions and excess chromosome fragments (deletions), Figure S6. Chromosome aberration frequencies generally failed to cluster patients, Figure S7. Processing of chromosome aberration data for XGBoost models, Table S1. Examples of individual telomere length data matrices used to train XGBoost models, Table S2. Metrics of XGBoost models for predicting post-IMRT telomeric outcomes, Table S3. Examples of chromosome aberration data matrices used to train XGBoost models, Table S4. Metrics of trained XGBoost models for predicting post-IMRT chromosome aberration frequencies.

## References

[1] Barnett G.C., West C.M.L., Dunning A.M., Elliott R.M., Coles C.E., Pharoah P.D.P., Burnet N.G. (2009). Normal Tissue Reactions to Radiotherapy. Nat. Rev. Cancer.

[2] Bentzen S.M. (2006). Preventing or Reducing Late Side Effects of Radiation Therapy: Radiobiology Meets Molecular Pathology. Nat. Rev. Cancer.

[3] Yusuf S.W., Venkatesulu B.P., Mahadevan L.S., Krishnan S. (2017). Radiation-Induced Cardiovascular Disease: A Clinical Perspective. Front. Cardiovasc. Med..

[4] Carver J.R., Shapiro C.L., Ng A., Jacobs L., Schwartz C., Virgo K.S., Hagerty K.L., Somerfield M.R., Vaughn D.J., ASCO Cancer Survivorship Expert Panel (2007). American Society of Clinical Oncology Clinical Evidence Review on the Ongoing Care of Adult Cancer Survivors: Cardiac and Pulmonary Late Effects. J. Clin. Oncol..

[5] Greene-Schloesser D., Robbins M.E. (2012). Radiation-Induced Cognitive Impairment-from Bench to Bedside. Neuro Oncol..

[6] Yaprak G., Gemici C., Temizkan S., Ozdemir S., Dogan B.C., Seseogullari O.O. (2018). Osteoporosis Development and Vertebral Fractures after Abdominal Irradiation in Patients with Gastric Cancer. BMC Cancer.

[7] Suit H., Goldberg S., Niemierko A., Ancukiewicz M., Hall E., Goitein M., Wong W., Paganetti H. (2007). Secondary Carcinogenesis in Patients Treated with Radiation: A Review of Data on Radiation-Induced Cancers in Human, Non-Human Primate, Canine and Rodent Subjects. Radiat. Res..

[8] Armstrong G.T., Stovall M., Robison L.L. (2010). Long-Term Effects of Radiation Exposure among Adult Survivors of Childhood Cancer: Results from the Childhood Cancer Survivor Study. Radiat. Res..

[9] Rajaraman P., Hauptmann M., Bouffler S., Wojcik A. (2018). Human Individual Radiation Sensitivity and Prospects for Prediction. Ann. ICRP.

[10] Habash M., Bohorquez L.C., Kyriakou E., Kron T., Martin O.A., Blyth B.J. (2017). Clinical and Functional Assays of Radiosensitivity and Radiation-Induced Second Cancer. Cancers.

[11] Banáth J.P., MacPhail S.H., Olive P.L. (2004). Radiation Sensitivity, H2AX Phosphorylation, and Kinetics of Repair of DNA Strand Breaks in Irradiated Cervical Cancer Cell Lines. Cancer Res..

[12] Redon C.E., Dickey J.S., Bonner W.M., Sedelnikova O.A. (2009). γ-H2AX as a Biomarker of DNA Damage Induced by Ionizing Radiation in Human Peripheral Blood Lymphocytes and Artificial Skin. Adv. Space Res..

[13] Schmitz A., Bayer J., Dechamps N., Goldin L., Thomas G. (2007). Heritability of Susceptibility to Ionizing Radiation-Induced Apoptosis of Human Lymphocyte Subpopulations. Int. J. Radiat. Oncol. Biol. Phys..

[14] Baeyens A., Thierens H., Claes K., Poppe B., Messiaen L., De Ridder L., Vral A. (2002). Chromosomal Radiosensitivity in Breast Cancer Patients with a Known or Putative Genetic Predisposition. Br. J. Cancer.

[15] Baria K., Warren C., Roberts S.A., West C.M., Scott D. (2001). Chromosomal Radiosensitivity as a Marker of Predisposition to Common Cancers?. Br. J. Cancer.

[16] Huber R., Braselmann H., Geinitz H., Jaehnert I., Baumgartner A., Thamm R., Figel M., Molls M., Zitzelsberger H. (2011). Chromosomal Radiosensitivity and Acute Radiation Side Effects after Radiotherapy in Tumour Patients—A Follow-up Study. Radiat. Oncol..

[17] Kerns S.L., Dorling L., Fachal L., Bentzen S., Pharoah P.D.P., Barnes D.R., Gómez-Caamaño A., Carballo A.M., Dearnaley D.P., Peleteiro P. (2016). Meta-Analysis of Genome Wide Association Studies Identifies Genetic Markers of Late Toxicity Following Radiotherapy for Prostate Cancer. EBioMedicine.

[18] Kerns S.L., Ostrer H., Stock R., Li W., Moore J., Pearlman A., Campbell C., Shao Y., Stone N., Kusnetz L. (2010). Genome-Wide Association Study to Identify Single Nucleotide Polymorphisms (SNPs) Associated with the Development of Erectile Dysfunction in African-American Men after Radiotherapy for Prostate Cancer. Int. J. Radiat. Oncol. Biol. Phys..

[19] Young A., Berry R., Holloway A.F., Blackburn N.B., Dickinson J.L., Skala M., Phillips J.L., Brettingham-Moore K.H. (2014). RNA-Seq Profiling of a Radiation Resistant and Radiation Sensitive Prostate Cancer Cell Line Highlights Opposing Regulation of DNA Repair and Targets for Radiosensitization. BMC Cancer.

[20] Bodalal Z., Trebeschi S., Nguyen-Kim T.D.L., Schats W., Beets-Tan R. (2019). Radiogenomics: Bridging Imaging and Genomics. Abdom. Radiol..

[21] Mirjolet C., Boidot R., Saliques S., Ghiringhelli F., Maingon P., Créhange G. (2015). The Role of Telomeres in Predicting Individual Radiosensitivity of Patients with Cancer in the Era of Personalized Radiotherapy. Cancer Treat. Rev..

[22] De Lange T. (2009). How Telomeres Solve the End-Protection Problem. Science.

[23] Moyzis R.K., Buckingham J.M., Cram L.S., Dani M., Deaven L.L., Jones M.D., Meyne J., Ratliff R.L., Wu J.R. (1988). A Highly Conserved Repetitive DNA Sequence, (TTAGGG)n, Present at the Telomeres of Human Chromosomes. Proc. Natl. Acad. Sci. USA.

[24] Von Zglinicki T. (2000). Role of Oxidative Stress in Telomere Length Regulation and Replicative Senescence. Ann. N. Y. Acad. Sci..

[25] Aubert G., Lansdorp P.M. (2008). Telomeres and Aging. Physiol. Rev..

[26] Vidaček N.Š., Nanić L., Ravlić S., Sopta M., Gerić M., Gajski G., Garaj-Vrhovac V., Rubelj I. (2017). Telomeres, Nutrition, and Longevity: Can We Really Navigate Our Aging?. J. Gerontol. Ser. A.

[27] Arsenis N.C., You T., Ogawa E.F., Tinsley G.M., Zuo L. (2017). Physical Activity and Telomere Length: Impact of Aging and Potential Mechanisms of Action. Oncotarget.

[28] Epel E.S., Blackburn E.H., Lin J., Dhabhar F.S., Adler N.E., Morrow J.D., Cawthon R.M. (2004). Accelerated Telomere Shortening in Response to Life Stress. Proc. Natl. Acad. Sci. USA.

[29] Miri M., Nazarzadeh M., Alahabadi A., Ehrampoush M.H., Rad A., Lotfi M.H., Sheikhha M.H., Sakhvidi M.J.Z., Nawrot T.S., Dadvand P. (2019). Air Pollution and Telomere Length in Adults: A Systematic Review and Meta-Analysis of Observational Studies. Environ. Pollut..

[30] Stout G.J., Blasco M.A. (2013). Telomere Length and Telomerase Activity Impact the UV Sensitivity Syndrome Xeroderma Pigmentosum C. Cancer Res..

[31] Broer L., Codd V., Nyholt D.R., Deelen J., Mangino M., Willemsen G., Albrecht E., Amin N., Beekman M., De Geus E.J.C. (2013). Meta-Analysis of Telomere Length in 19 713 Subjects Reveals High Heritability, Stronger Maternal Inheritance and a Paternal Age Effect. Eur. J. Hum. Genet..

[32] Delgado D.A., Zhang C., Gleason K., Demanelis K., Chen L.S., Gao J., Roy S., Shinkle J., Sabarinathan M., Argos M. (2019). The Contribution of Parent-to-Offspring Transmission of Telomeres to the Heritability of Telomere Length in Humans. Hum. Genet..

[33] Honig L.S., Kang M.S., Cheng R., Eckfeldt J.H., Thyagarajan B., Leiendecker-Foster C., Province M.A., Sanders J.L., Perls T., Christensen K. (2015). Heritability of Telomere Length in a Study of Long-Lived Families. Neurobiol. Aging.

[34] Weng Q., Du J., Yu F., Huang T., Chen M., Lv H., Ma H., Hu Z., Jin G., Hu Y. (2016). The Known Genetic Loci for Telomere Length May Be Involved in the Modification of Telomeres Length after Birth. Sci. Rep..

[35] Ayouaz A., Raynaud C., Heride C., Revaud D., Sabatier L. (2008). Telomeres: Hallmarks of Radiosensitivity. Biochimie.

[36] Berardinelli F., Antoccia A., Buonsante R., Gerardi S., Cherubini R., Nadal V.D., Tanzarella C., Sgura A. (2013). The Role of Telomere Length Modulation in Delayed Chromosome Instability Induced by Ionizing Radiation in Human Primary Fibroblasts. Environ. Mol. Mutagenesis.

[37] Maeda T., Nakamura K., Atsumi K., Hirakawa M., Ueda Y., Makino N. (2013). Radiation-Associated Changes in the Length of Telomeres in Peripheral Leukocytes from Inpatients with Cancer. Int. J. Radiat. Biol..

[38] Sgura A., Antoccia A., Berardinelli F., Cherubini R., Gerardi S., Zilio C., Tanzarella C. (2006). Telomere Length in Mammalian Cells Exposed to Low- and High-LET Radiations. Radiat. Prot. Dosim..

[39] Haycock P.C., Burgess S., Nounu A., Zheng J., Okoli G.N., Bowden J., Wade K.H., Timpson N.J., Evans D.M., Telomeres Mendelian Randomization Collaboration (2017). Association Between Telomere Length and Risk of Cancer and Non-Neoplastic Diseases: A Mendelian Randomization Study. JAMA Oncol..

[40] Protsenko E., Rehkopf D., Prather A.A., Epel E., Lin J. (2020). Are Long Telomeres Better than Short? Relative Contributions of Genetically Predicted Telomere Length to Neoplastic and Non-Neoplastic Disease Risk and Population Health Burden. PLoS ONE.

[41] Stone R.C., Horvath K., Kark J.D., Susser E., Tishkoff S.A., Aviv A. (2016). Telomere Length and the Cancer–Atherosclerosis Trade-Off. PLoS Genet..

[42] Armanios M., Blackburn E.H. (2012). The Telomere Syndromes. Nat. Rev. Genet..

[43] Martínez P., Blasco M.A. (2018). Heart-Breaking Telomeres. Circ. Res..

[44] Calado R.T., Cooper J.N., Padilla-Nash H.M., Sloand E.M., Wu C.O., Scheinberg P., Ried T., Young N.S. (2012). Short Telomeres Result in Chromosomal Instability in Hematopoietic Cells and Precede Malignant Evolution in Human Aplastic Anemia. Leukemia.

[45] Adams M.J., Hardenbergh P.H., Constine L.S., Lipshultz S.E. (2003). Radiation-Associated Cardiovascular Disease. Crit. Rev. Oncol. Hematol..

[46] Green D.E., Rubin C.T. (2014). Consequences of Irradiation on Bone and Marrow Phenotypes, and Its Relation to Disruption of Hematopoietic Precursors. Bone.

[47] Tsoutsou P.G., Koukourakis M.I. (2006). Radiation Pneumonitis and Fibrosis: Mechanisms Underlying Its Pathogenesis and Implications for Future Research. Int. J. Radiation Oncol.* Biol.* Phys..

[48] McNally E.J., Luncsford P.J., Armanios M. (2019). Long Telomeres and Cancer Risk: The Price of Cellular Immortality. J. Clin. Investig..

[49] Dracham C.B., Shankar A., Madan R. (2018). Radiation Induced Secondary Malignancies: A Review Article. Radiat. Oncol. J..

[50] Bains S.K., Chapman K., Bright S., Senan A., Kadhim M., Slijepcevic P. (2019). Effects of Ionizing Radiation on Telomere Length and Telomerase Activity in Cultured Human Lens Epithelium Cells. Int. J. Radiat. Biol..

[51] Berardinelli F., Sgura A., Di Masi A., Leone S., Cirrone G.A.P., Romano F., Tanzarella C., Antoccia A. (2014). Radiation-Induced Telomere Length Variations in Normal and in Nijmegen Breakage Syndrome Cells. Int. J. Radiat. Biol..

[52] De Vitis M., Berardinelli F., Coluzzi E., Marinaccio J., O’Sullivan R.J., Sgura A. (2019). X-Rays Activate Telomeric Homologous Recombination Mediated Repair in Primary Cells. Cells.

[53] Alsner J., Rødningen O.K., Overgaard J. (2007). Differential Gene Expression before and after Ionizing Radiation of Subcutaneous Fibroblasts Identifies Breast Cancer Patients Resistant to Radiation-Induced Fibrosis. Radiother. Oncol..

[54] Andreassen C.N., Overgaard J., Alsner J. (2013). Independent Prospective Validation of a Predictive Test for Risk of Radiation Induced Fibrosis Based on the Gene Expression Pattern in Fibroblasts Irradiated in Vitro. Radiother. Oncol..

[55] Borgmann K., Hoeller U., Nowack S., Bernhard M., Röper B., Brackrock S., Petersen C., Szymczak S., Ziegler A., Feyer P. (2008). Individual Radiosensitivity Measured with Lymphocytes May Predict the Risk of Acute Reaction after Radiotherapy. Int. J. Radiat. Oncol. Biol. Phys..

[56] Paul S., Barker C.A., Turner H.C., McLane A., Wolden S.L., Amundson S.A. (2011). Prediction of In Vivo Radiation Dose Status in Radiotherapy Patients Using Ex Vivo and In Vivo Gene Expression Signatures. Radiat. Res..

[57] Huang L., Snyder A.R., Morgan W.F. (2003). Radiation-Induced Genomic Instability and Its Implications for Radiation Carcinogenesis. Oncogene.

[58] Willis N.A., Rass E., Scully R. (2015). Deciphering the Code of the Cancer Genome: Mechanisms of Chromosome Rearrangement. Trends Cancer.

[59] Ward J.F., Cohn W.E., Moldave K. (1988). DNA Damage Produced by Ionizing Radiation in Mammalian Cells: Identities, Mechanisms of Formation, and Reparability. Progress in Nucleic Acid Research and Molecular Biology.

[60] Behjati S., Gundem G., Wedge D.C., Roberts N.D., Tarpey P.S., Cooke S.L., Van Loo P., Alexandrov L.B., Ramakrishna M., Davies H. (2016). Mutational Signatures of Ionizing Radiation in Second Malignancies. Nat. Commun..

[61] Mosesso P., Cinelli S., Dhawan A., Bajpayee M. (2019). In Vitro Cytogenetic Assays: Chromosomal Aberrations and Micronucleus Tests. Genotoxicity Assessment: Methods and Protocols.

[62] Ray F.A., Zimmerman E., Robinson B., Cornforth M.N., Bedford J.S., Goodwin E.H., Bailey S.M. (2013). Directional Genomic Hybridization for Chromosomal Inversion Discovery and Detection. Chromosome Res..

[63] Pella A., Cambria R., Riboldi M., Jereczek-Fossa B.A., Fodor C., Zerini D., Torshabi A.E., Cattani F., Garibaldi C., Pedroli G. (2011). Use of Machine Learning Methods for Prediction of Acute Toxicity in Organs at Risk Following Prostate Radiotherapy. Med. Phys..

[64] Fan J., Wang J., Chen Z., Hu C., Zhang Z., Hu W. (2019). Automatic Treatment Planning Based on Three-Dimensional Dose Distribution Predicted from Deep Learning Technique. Med. Phys..

[65] Lee S., Liang X., Woods M., Reiner A.S., Concannon P., Bernstein L., Lynch C.F., Boice J.D., Deasy J.O., Bernstein J.L. (2020). Machine Learning on Genome-Wide Association Studies to Predict the Risk of Radiation-Associated Contralateral Breast Cancer in the WECARE Study. PLoS ONE.

[66] Chen T., Guestrin C. XGBoost: A Scalable Tree Boosting System. Proceedings of the 22nd ACM SIGKDD International Conference on Knowledge Discovery and Data Mining—KDD ’16.

[67] Taninaga J., Nishiyama Y., Fujibayashi K., Gunji T., Sasabe N., Iijima K., Naito T. (2019). Prediction of Future Gastric Cancer Risk Using a Machine Learning Algorithm and Comprehensive Medical Check-up Data: A Case-Control Study. Sci. Rep..

[68] Yu D., Liu Z., Su C., Han Y., Duan X., Zhang R., Liu X., Yang Y., Xu S. (2020). Copy Number Variation in Plasma as a Tool for Lung Cancer Prediction Using Extreme Gradient Boosting (XGBoost) Classifier. Thorac. Cancer.

[69] Wang J., Yang P., Zhao Y., Elhalawani H., Liu R., Zhu H., Mohamed A.S., Fuller C.D., Zhu H. (2019). A Predictive Model of Radiation-Related Fibrosis Based on Radiomic Features of Magnetic Resonance Imaging. Int. J. Radiat. Oncol. Biol. Phys..

[70] Poon S.S.S., Lansdorp P.M. (2001). Quantitative Fluorescence In Situ Hybridization (Q-FISH). Curr. Protoc. Cell Biol..

[71] Howe B., Umrigar A., Tsien F. (2014). Chromosome Preparation From Cultured Cells. J. Vis. Exp..

[72] Williams E.S., Bailey S.M. (2009). Chromosome Orientation Fluorescence in Situ Hybridization (CO-FISH). Cold Spring Harb. Protoc..

[73] Schneider C.A., Rasband W.S., Eliceiri K.W. (2012). NIH Image to ImageJ: 25 Years of Image Analysis. Nat. Methods.

[74] Wong H.-P., Slijepcevic P. (2004). Telomere Length Measurement in Mouse Chromosomes by a Modified Q-FISH Method. CGR.

[75] Seabold S., Perktold J. Statsmodels: Econometric and Statistical Modeling with Python. Proceedings of the 9th Python in Science Conference.

[76] Virtanen P., Gommers R., Oliphant T.E., Haberland M., Reddy T., Cournapeau D., Burovski E., Peterson P., Weckesser W., Bright J. (2020). SciPy 1.0: Fundamental Algorithms for Scientific Computing in Python. Nat. Methods.

[77] Pedregosa F., Varoquaux G., Gramfort A., Michel V., Thirion B., Grisel O., Blondel M., Müller A., Nothman J., Louppe G. (2018). Scikit-Learn: Machine Learning in Python. arXiv.

[78] Garrett-Bakelman F.E., Darshi M., Green S.J., Gur R.C., Lin L., Macias B.R., McKenna M.J., Meydan C., Mishra T., Nasrini J. (2019). The NASA Twins Study: A Multidimensional Analysis of a Year-Long Human Spaceflight. Science.

[79] Luxton J.J., McKenna M.J., Lewis A., Taylor L.E., George K.A., Dixit S.M., Moniz M., Benegas W., Mackay M.J., Mozsary C. (2020). Telomere Length Dynamics and DNA Damage Responses Associated with Long-Duration Spaceflight. Cell Rep..

[80] Bergstra J., Bengio Y. (2012). Random Search for Hyper-Parameter Optimization. J. Mach. Learn. Res..

[81] Stone M. (1974). Cross-Validatory Choice and Assessment of Statistical Predictions. J. R. Stat. Soc. Ser. B (Methodol.).

[82] Cornforth M.N., Anur P., Wang N., Robinson E., Ray F.A., Bedford J.S., Loucas B.D., Williams E.S., Peto M., Spellman P. (2018). Molecular Cytogenetics Guides Massively Parallel Sequencing of a Radiation-Induced Chromosome Translocation in Human Cells. Radiat. Res..

[83] Littlefield L.G., Colyer S.P., Joiner E.E., DuFrain R.J. (1979). Sister Chromatid Exchanges in Human Lymphocytes Exposed to Ionizing Radiation during G0. Radiat. Res..

[84] Morgan W.F., Crossen P.E. (1980). X Irradiation and Sister Chromatid Exchange in Cultured Human Lymphocytes. Environ. Mutagen..

[85] Ballarini F., Altieri S., Bortolussi S., Carante M., Giroletti E., Protti N. (2015). The Role of DNA Cluster Damage and Chromosome Aberrations in Radiation-Induced Cell Killing: A Theoretical Approach. Radiat. Prot. Dosim..

[86] Carrano A.V. (1973). Chromosome Aberrations and Radiation-Induced Cell Death: II. Predicted and Observed Cell Survival. Mutat. Res./Fundam. Mol. Mech. Mutagen..

