# Supplementary material for: Telomere Length Dynamics and Chromosomal Instability for Predicting Individual Radiosensitivity and Risk via Machine Learning
Source: J Pers Med. 2021 Mar 8;11(3):188. doi: 10.3390/jpm11030188 (PMC8002073; doi:10.3390/jpm11030188)
Supplement: Supplementary file 1 [file jpm-11-00188-s001.pdf]

Supplemental Figure Legends

Figure S1. Correlations between telomere length, peripheral white blood cells, and lymphocytes. A. Mean telomere length (Telo-FISH) plotted longitudinally against peripheral white blood cell (WBC) counts (thousands per microliter) from complete blood count tests for all patients: 1 non irrad = pre-IMRT non-irradiated (0 Gy); 2 irrad @ 4 Gy = pre-IMRT *in vitro* irradiated; 3B = immediate post-IMRT; and 4C = 3-month post-IMRT. Dark lines denote medians, lighter bands denote confidence intervals. Pearson correlation R^2 values were calculated on a per patient basis. **B.** Correlations between mean telomere length and five main WBC types, and **C.** proportions of lymphocyte cell types.

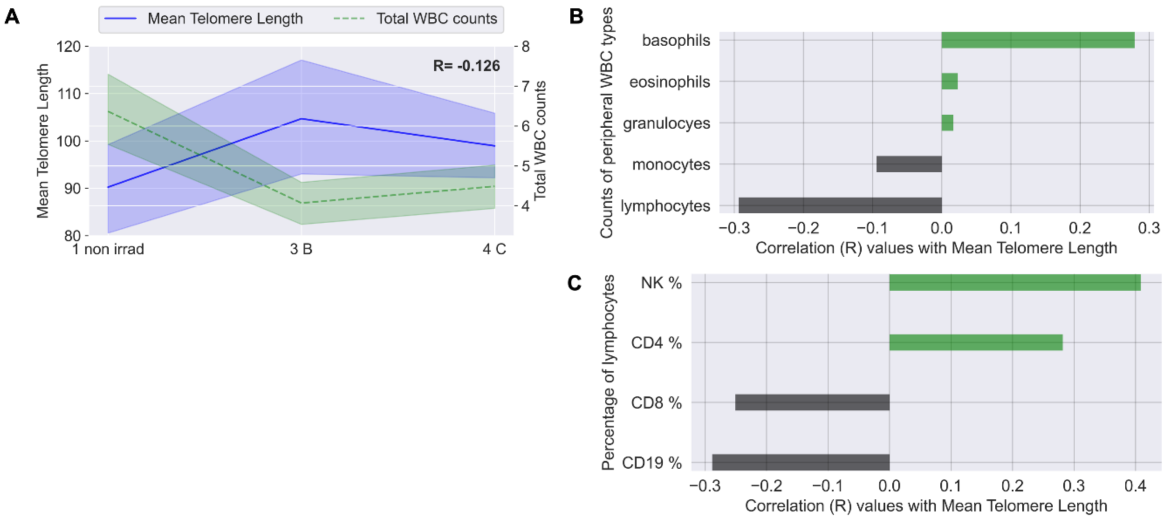

Figure S2. Strong generalizability of XGBoost models to new patient data (leave one out). A – M. Fourteen separate XGBoost models were iteratively trained on pre-IMRT individual telomere length measurements (n=93,840, Telo-FISH), excluding one patient, and tested to predict 3-month post-IMRT mean telomere length, with the inclusion of the patient excluded during training. Each panel is one model; patients excluded during training for that model are noted in the panel headers and plotted in black. Dark lines represent a simple regression line (X/Y), lighter bands the 95% confidence interval, R^2 values (coefficient of determination) are noted in bold.

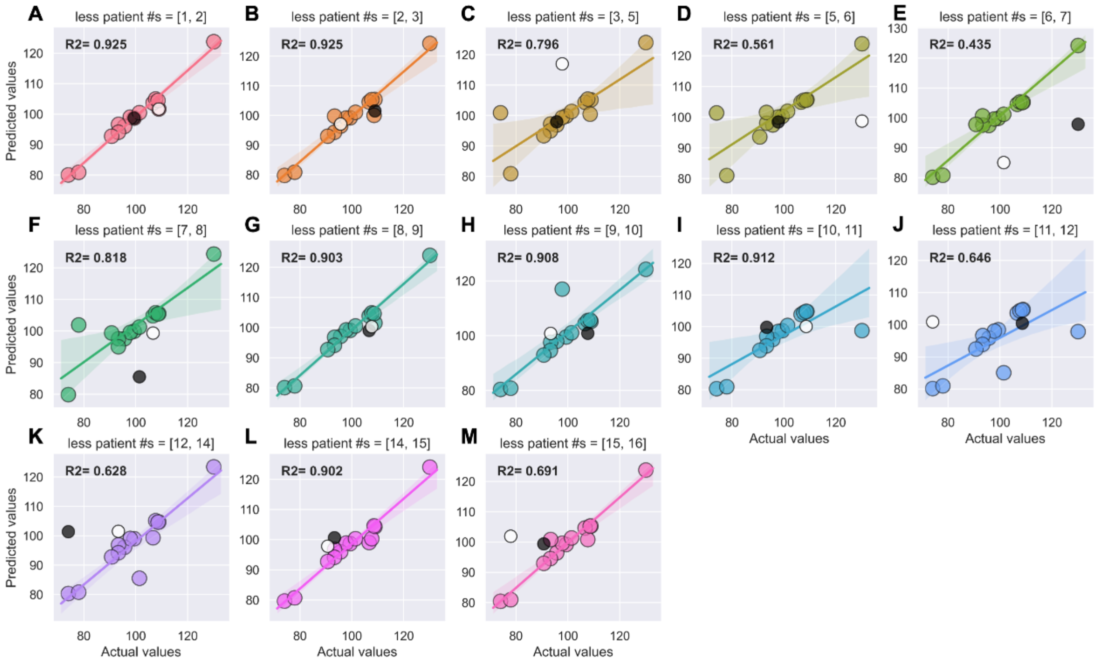

Figure S3. Strong generalizability of XGBoost models to new patient data (leave two out). A – L. Thirteen separate XGBoost models were iteratively trained on pre-IMRT individual telomere length measurements (n=84,640, Telo-FISH), excluding two patients, and tested to predict 3-month post-IMRT mean telomere length, with the inclusion of the two patients excluded during training. Each panel is one model; patients excluded during training for that model are noted in the panel headers and plotted in (black: index 1, white: index 2). Dark lines represent a simple regression line (X/Y), lighter bands the 95% confidence interval, R^2 values (coefficient of determination) are noted in bold.

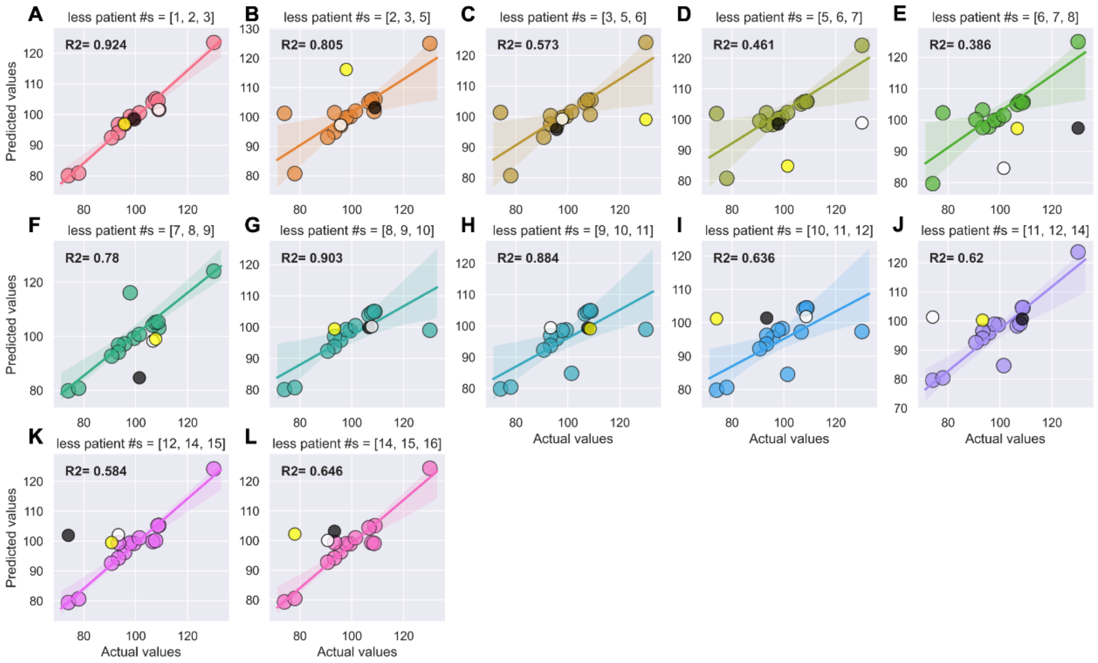

Figure S4. Correlations between chromosome aberrations and peripheral blood lymphocytes. Average frequencies of chromosome aberrations plotted longitudinally against lymphocyte cell counts (thousands per microliter) from complete blood count tests for all patients: 1 non irrad = pre-IMRT non-irradiated (0 Gy); 2 irrad @ 4 Gy = pre-IMRT *in vitro* irradiated; 3B = immediate post-IMRT; and 4C = 3-month post-IMRT. **A**. Inversions, **B**. translocations, **C**. dicentrics, and **D**. excess chromosome fragments (deletions) and lymphocyte cell counts. Center lines denote medians, lighter bands denote confidence intervals. Pearson correlation R^2 values were calculated between plotted values on a per patient basis and noted in bold on each graph.

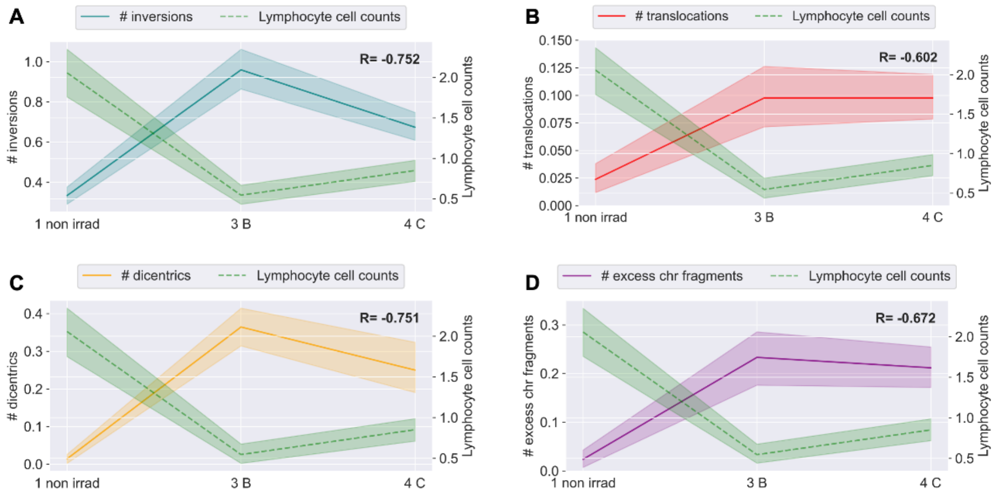

Figure S5. Clustering of patients by inversions and excess chromosome fragments (deletions). Hierarchical clustering of patients by longitudinal changes in chromosome aberrations scored by directional Genomic Hybridization (dGH): 1 non irrad = pre-IMRT non-irradiated (0 Gy); 2 irrad @ 4 Gy = pre-IMRT *in vitro* irradiated; 3B = immediate post-IMRT; and 4C = 3-month post-IMRT. Patients were clustered by **A.** inversions, and **B.** excess chromosome fragments (z-score normalized). Patient ID 13 not clustered (sample failed to culture).

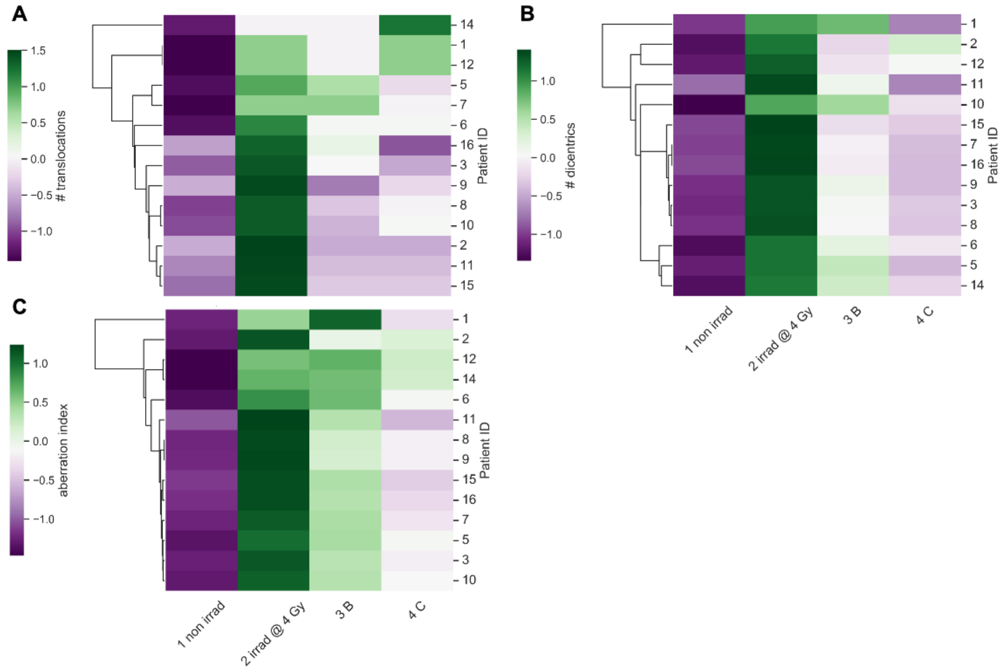

Figure S6. Chromosome aberrations generally failed to cluster patients. Hierarchical clustering of patients by longitudinal changes in chromosome aberrations scored by directional Genomic Hybridization (dGH): 1 non irrad = pre-IMRT non-irradiated (0 Gy); 2 irrad @ 4 Gy = pre-IMRT *in vitro* irradiated; 3B = immediate post-IMRT; and 4C = 3-month post-IMRT. Patients were clustered by **A.** translocations, **B.** dicentrics, and **C.** aberration index (z-score normalized). Aberration index was created by summing all aberrations (inversions, translocations, dicentrics, excess chromosome fragments) per cell. Patient ID 13 not clustered (sample failed to culture).

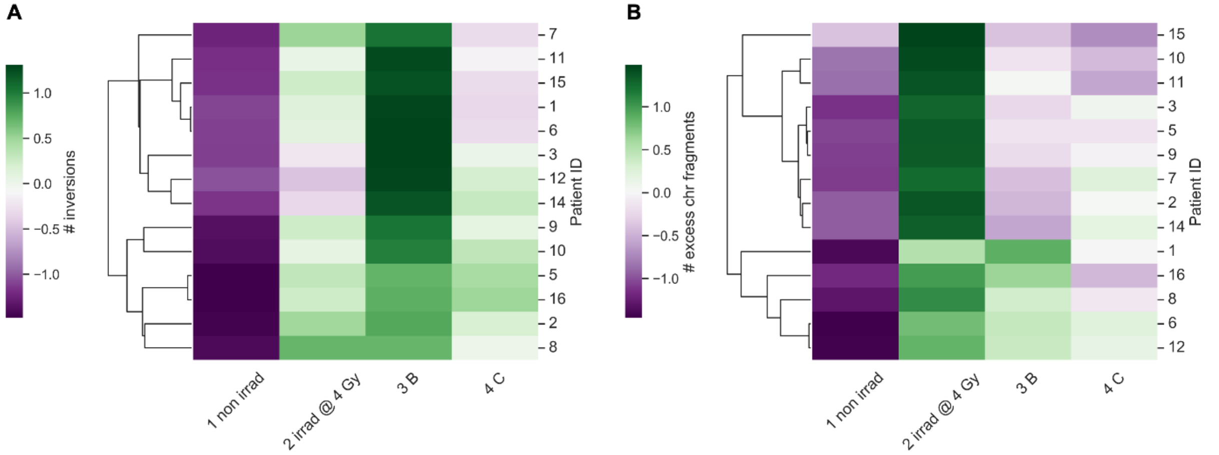

Figure S7. Processing of chromosome aberration data for XGBoost models. Schematic for machine learning pipeline using chromosome aberration (CA) data. Preprocessed data: Feature 1: pre-IMRT counts of scored CAs; Feature 2: pre-IMRT sample labels (non-irradiated, *in vitro* irradiated, encoded as 0/1); Feature n: represents pre-IMRT counts of multiple types of CAs (for aberration index). Target: Late post-IMRT average frequencies of CAs (either specific aberration type or aberration index). Data is randomly shuffled and stratified (by patient ID and pre-IMRT sample origin) and split into training (80%) and testing (20%) datasets; patient IDs are stripped after splitting. Five-fold cross validation was used, and models were evaluated with Mean Absolute Error (MAE) and R^2 between predicted and true values in the test set. See Materials and Methods and Code availability for model parameters and implementations in Python.

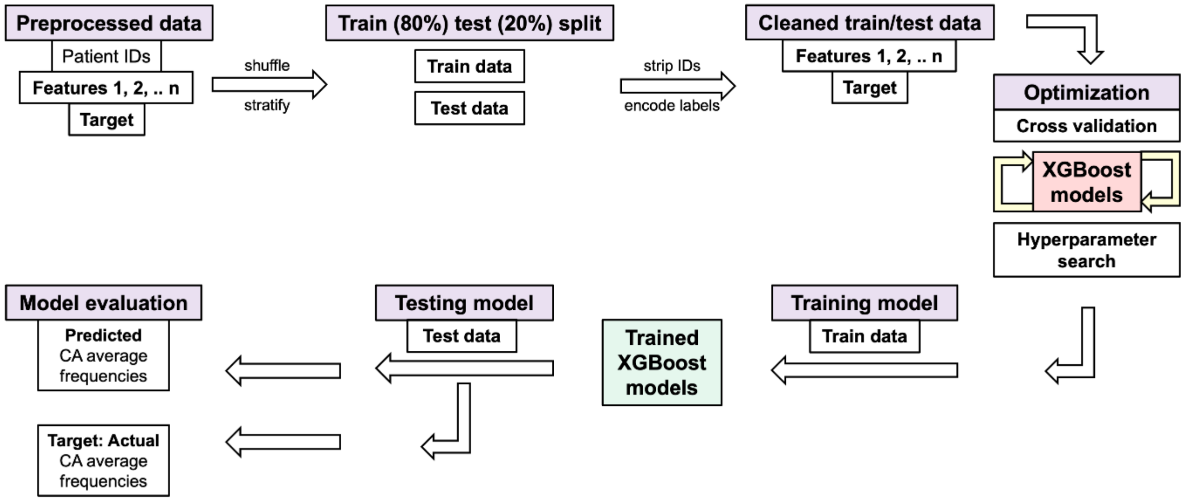

Table S1. Examples of individual telomere length data matrices used to train XGBoost models. XGBoost models were trained on 103,040 individual telomere length measurements (one telomere per row) (Telo-FISH) from pre-IMRT non-irradiated (1 non irrad, 0 Gy) and *in vitro* irradiated (2 irrad @ 4 Gy) samples to predict 3-month post-IMRT (4C) telomeric outcomes. Matrices represent examples of pre- (**A,C,E**) and post-processed (**B,D,F**) training data. Patient IDs are stripped after data is shuffled and stratified. The 'encoded sample origin' column contains numerical encodings denoting individual telomeres' pre-IMRT sample of origin (0: non-irradiated, 1: in vitro irradiated). XGBoost models were trained to predict mean telomere length (**A,B**) and numbers of short (**C,D**) and long (**E,F**) telomeres at 3-month post-IMRT with data in the format as shown.

A

patient id	pre-therapy sample origin	individual telomeres (RFI)	4 C telo means
1	1 non irrad	52.79329603949808	99.34629891451401
1	2 irrad @ 4 Gy	100.30726247504634	99.34629891451401
1	1 non irrad	59.12849156423784	99.34629891451401
1	2 irrad @ 4 Gy	106.64139157520613	99.34629891451401
1	1 non irrad	69.68715077213746	99.34629891451401
1	2 irrad @ 4 Gy	107.69724693733689	99.34629891451401

B

encoded sample origin	individual telomeres (RFI)	4 C telo means
1.0	71.84704355757034	90.6803515449468
0.0	58.01948086775996	108.91532697997721
0.0	125.05216035895008	93.35225326745208
0.0	99.84003125432304	93.35225326745208
0.0	157.34096506511176	108.91532697997721
1.0	59.127900279322205	99.34629891451401

C

patient id	pre-therapy sample origin	individual telomeres (RFI)	4 C # short telos
1	1 non irrad	52.79329603949808	372
1	2 irrad @ 4 Gy	100.30726247504634	372
1	1 non irrad	59.12849156423784	372
1	2 irrad @ 4 Gy	106.64139157520613	372
1	1 non irrad	69.68715077213746	372
1	2 irrad @ 4 Gy	107.69724693733689	372

D

encoded sample origin	individual telomeres (RFI)	4 C # short telos
0.0	39.80714575487005	319.0
0.0	84.7669312523909	2028.0
0.0	48.569832356338225	372.0
1.0	99.34779587017763	829.0
1.0	104.85784735429183	319.0
1.0	92.25878757956735	124.0

E

patient id	pre-therapy sample origin	individual telomeres (RFI)	4 C # long telos
1	1 non irrad	52.79329603949808	1987
1	2 irrad @ 4 Gy	100.30726247504634	1987
1	1 non irrad	59.12849156423784	1987
1	2 irrad @ 4 Gy	106.64139157520613	1987
1	1 non irrad	69.68715077213746	1987
1	2 irrad @ 4 Gy	107.69724693733689	1987

F

encoded sample origin	individual telomeres (RFI)	4 C # long telos
0.0	56.551567152220926	2026.0
0.0	103.18180673387677	2026.0
0.0	69.58478047400733	365.0
0.0	56.18104859876975	1078.0
1.0	137.72889825629034	1002.0
1.0	84.46927366319693	1987.0

Table S2. Metrics of XGBoost models for predicting post-IMRT telomeric outcomes. XGBoost models were trained on pre-IMRT individual telomere length measurements (Telo-FISH) to predict 3-month post-IMRT telomeric outcomes. Metrics assess model performance during (five) cross-fold validation (CV) (columns 1-2 from left), and when challenged with the test set (test) (columns 3-4 from left). Model performance was evaluated with mean absolute error (MAE) (std dev: standard deviation) across a range of samples in the training data (n=100 to 103,040). R^2: correlation metric. Metrics of XGBoost models for predicting 3-month post-IMRT (4C) mean telomere length **A**, numbers of short **B**, and long **C** telomeres.

A

Average MAE of CV folds	Std dev of MAE of CV folds	MAE predicted vs. test values	R2 predicted vs. test values	N samples training data
11.4602	1.6502	13.4903	-0.8393	100.0
10.6657	0.4454	10.3646	-0.2049	500.0
8.0423	0.486	7.9009	0.1788	1000.0
6.7089	0.3895	6.0449	0.5126	2000.0
4.8488	0.2224	4.642	0.7094	4000.0
3.9282	0.0988	3.7677	0.8215	8000.0
3.6385	0.0447	3.5413	0.851	16000.0
3.3792	0.0626	3.3483	0.8755	32000.0
3.2944	0.051	3.2521	0.881	64000.0
3.233	0.052	3.2596	0.8817	103040.0

B

Average MAE of CV folds	Std dev of MAE of CV folds	MAE predicted vs. test values	R2 predicted vs. test values	N samples training data
705.0956	48.4789	680.9499	-0.5887	100.0
573.3922	25.5422	521.0982	-0.0162	500.0
440.9283	22.7264	425.5251	0.2572	1000.0
366.4338	19.0126	326.1635	0.5396	2000.0
315.2925	5.9607	292.0579	0.6593	4000.0
269.2991	6.6633	260.4209	0.7433	8000.0
257.6623	3.5097	247.6769	0.7747	16000.0
243.5729	4.1386	241.8505	0.7987	32000.0
233.7408	5.251	231.1663	0.803	64000.0
236.2825	2.0593	234.1744	0.8112	103040.0

C

Average MAE of CV folds	Std dev of MAE of CV folds	MAE predicted vs. test values	R2 predicted vs. test values	N samples training data
1056.6558	219.1554	953.2471	-0.4405	100.0
763.7998	38.9092	727.2706	0.0447	500.0
629.6607	49.9928	627.9304	0.2945	1000.0
548.353	24.8756	481.5782	0.5641	2000.0
409.3232	4.8234	415.0895	0.674	4000.0
382.1325	11.974	376.8821	0.7505	8000.0
353.0249	6.234	348.5064	0.7981	16000.0
343.0401	4.5386	329.2967	0.8128	32000.0
331.0765	3.7999	331.8519	0.813	64000.0
330.3521	2.0857	335.931	0.8191	103040.0

Table S3. Examples of chromosome aberration data matrices used to train XGBoost models. XGBoost models were trained on chromosome aberration count data (one cell per row, n=672) from pre-IMRT non-irradiated (1 non irrad, 0 Gy) and *in vitro* irradiated (2 irrad @ 4 Gy) samples to predict 3month post-IMRT (4C) chromosome aberration frequencies. Matrices represent pre- (**A,C**) and post-processed (**B,D**) training data. Patient IDs are stripped after data is shuffled and stratified. The 'encoded sample origin' column contains numerical encodings denoting cells' pre-IMRT sample of origin (0: non-irradiated, 1: *in vitro* irradiated). XGBoost models shown were trained to predict average inversion frequencies (**A,B**) and aberration index frequencies (**C,D**).

A

patient id	pre-therapy sample origin	# inversions	4 C # inversions
5	1 non irrad	0	0.7083333333333334
11	2 irrad @ 4 Gy	2	0.4583333333333333
1	1 non irrad	0	0.5
9	1 non irrad	0	0.7083333333333334
11	1 non irrad	0	0.4583333333333333
16	1 non irrad	0	0.7916666666666666

B

encoded sample origin	# inversions	4 C # inversions
0.0	0.0	0.7083333333333334
1.0	0.0	0.5
1.0	0.0	0.5
0.0	1.0	0.7083333333333334
1.0	0.0	0.5
1.0	1.0	0.7916666666666666

C

patient id	pre-therapy sample origin	# inversions	# translocations	# dicentrics	# excess chr fragments	4 C aberration index
9	1 non irrad	0	0	0	0	1.125
7	2 irrad @ 4 Gy	1	0	1	1	0.8333333333333334
11	2 irrad @ 4 Gy	0	1	0	0	0.6666666666666666
1	1 non irrad	0	0	0	0	0.9583333333333334
16	1 non irrad	0	0	0	0	1.0833333333333333
6	1 non irrad	0	0	0	0	1.375

D

encoded sample origin	# inversions	# translocations	# dicentrics	# excess chr fragments	4 C aberration index
0.0	0.0	0.0	0.0	0.0	1.0833333333333333
0.0	0.0	0.0	0.0	0.0	0.9583333333333334
1.0	0.0	0.0	0.0	0.0	1.4166666666666667
0.0	0.0	0.0	0.0	0.0	1.2083333333333333
1.0	2.0	0.0	0.0	0.0	1.2083333333333333
0.0	0.0	0.0	0.0	0.0	0.9166666666666666

Table S4. Metrics of trained XGBoost models for predicting post-IMRT average frequencies of chromosome aberrations. A-C. Multiple iterations of XGBoost models were trained on pre-IMRT chromosome aberration counts per cell (n=672 cells) to predict late post-IMRT average chromosome aberration frequencies. Time points for pre-IMRT data were encoded (0/1: non-irradiated, *in vitro* irradiated). Metrics assess model performance during (five) cross-fold validation (CV) and when challenged with the test set (test). Model performance was evaluated with mean absolute error (MAE) (std dev: standard deviation). R^2: correlation metric. Performance of models with identical initializations and hyperparameters for predicting average frequencies of inversions, translocations, dicentrics, chromosome fragments, and aberration index are shown.

A

Features	Target	Average MAE of CV folds	Std dev of MAE of CV folds	MAE predicted vs. test values	R2 predicted vs. test values
# inversions, encoded samples	4 C # inversions	0.1746	0.0445	0.2724	-0.213
# translocations, encoded samples	4 C # translocations	0.0412	0.0188	0.1327	-0.3905
# dicentrics, encoded samples	4 C # dicentrics	0.1171	0.0461	0.2508	0.0019
# excess chr fragments, encoded samples	4 C # excess chr fragments	0.0939	0.0334	0.1787	-0.1228
all aberrations, encoded samples	4 C aberration index	0.2541	0.0496	0.5137	-0.05

B

Features	Target	Average MAE of CV folds	Std dev of MAE of CV folds	MAE predicted vs. test values	R2 predicted vs. test values
# inversions, encoded samples	4 C # inversions	0.1759	0.0332	0.2187	-1.5965
# translocations, encoded samples	4 C # translocations	0.0375	0.0096	0.1096	-0.004
# dicentrics, encoded samples	4 C # dicentrics	0.1167	0.0463	0.2023	-0.0215
# excess chr fragments, encoded samples	4 C # excess chr fragments	0.084	0.0103	0.202	-0.1071
all aberrations, encoded samples	4 C aberration index	0.3294	0.1258	0.3596	-0.0256

C

Features	Target	Average MAE of CV folds	Std dev of MAE of CV folds	MAE predicted vs. test values	R2 predicted vs. test values
# inversions, encoded samples	4 C # inversions	0.15	0.0573	0.1977	-0.0709
# translocations, encoded samples	4 C # translocations	0.0448	0.0145	0.0977	-0.0346
# dicentrics, encoded samples	4 C # dicentrics	0.1123	0.034	0.2177	-0.0558
# excess chr fragments, encoded samples	4 C # excess chr fragments	0.0681	0.0155	0.194	-0.0372
all aberrations, encoded samples	4 C aberration index	0.3255	0.0528	0.5078	-0.0259
